# BRD9 defines a SWI/SNF sub-complex and constitutes a specific vulnerability in malignant rhabdoid tumors

**DOI:** 10.1038/s41467-019-09891-7

**Published:** 2019-04-23

**Authors:** Xiaofeng Wang, Su Wang, Emma C. Troisi, Thomas P. Howard, Jeffrey R. Haswell, Bennett K. Wolf, William H. Hawk, Pilar Ramos, Elaine M. Oberlick, Evgeni P. Tzvetkov, Aaron Ross, Francisca Vazquez, William C. Hahn, Peter J. Park, Charles W. M. Roberts

**Affiliations:** 1 Department of Molecular and Systems Biology, Geisel School of Medicine, Dartmouth College, Hanover, NH 03756 USA; 2 Department of Pediatric Oncology, Dana-Farber Cancer Institute, Boston, MA 02215 USA; 3 Department of Biomedical Informatics, Harvard Medical School, Boston, MA 02115 USA; 4 Broad Institute of Harvard and MIT, 415 Main Street, Cambridge, MA 02142 USA; 5 Comprehensive Cancer Center and Department of Oncology, St. Jude Children’s Research Hospital, Memphis, TN 38105 USA; 6 Department of Medical Oncology, Dana-Farber Cancer Institute, Boston, MA 02215 USA

**Keywords:** Biochemistry, Cancer, Epigenetics

## Abstract

Bromodomain-containing protein 9 (BRD9) is a recently identified subunit of SWI/SNF(BAF) chromatin remodeling complexes, yet its function is poorly understood. Here, using a genome-wide CRISPR-Cas9 screen, we show that BRD9 is a specific vulnerability in pediatric malignant rhabdoid tumors (RTs), which are driven by inactivation of the *SMARCB1* subunit of SWI/SNF. We find that BRD9 exists in a unique SWI/SNF sub-complex that lacks SMARCB1, which has been considered a core subunit. While SMARCB1-containing SWI/SNF complexes are bound preferentially at enhancers, we show that BRD9-containing complexes exist at both promoters and enhancers. Mechanistically, we show that SMARCB1 loss causes increased BRD9 incorporation into SWI/SNF thus providing insight into BRD9 vulnerability in RTs. Underlying the dependency, while its bromodomain is dispensable, the DUF3512 domain of BRD9 is essential for SWI/SNF integrity in the absence of SMARCB1. Collectively, our results reveal a BRD9-containing SWI/SNF subcomplex is required for the survival of *SMARCB1*-mutant RTs.

## Abstract (web summary)

Mutations of the SWI/SNF chromatin-remodeling complex member SMARCB1 can cause malignant rhaboid tumors. Here the authors report a BRD9-containing SWI/SNF subcomplex that lacks SMARCB1 and its requirement for the survival of rhaboid tumors.

## Introduction

SMARCB1 (also known as SNF5, INI1, and BAF47), a subunit of SWI/SNF chromatin-remodeling complexes^1,2^, is biallelically inactivated in the vast majority (95%) of malignant rhabdoid tumors (RTs)^3,4^, aggressive and lethal cancers that predominantly strike young children. These cancers arise most frequently in the kidney or brain but can arise in soft tissues throughout the body. RTs are often refractory to even intensive therapies and the majority of children die of their disease. Despite their aggressiveness, RTs are genetically simple and have one of the lowest mutation rates seen across all cancers^5,6^. SMARCB1 inactivation alters the transcription of many genes in a context dependent manner^1^, with mechanistic insight coming from recent studies that demonstrated a central role for SMARCB1 in the establishment and maintenance of in active enhancers^7–9^. SMARCB1 loss results in widespread impairment of typical enhancer activity while residual SWI/SNF complexes are relatively maintained at super-enhancers, leading to impaired transcriptional programs that underlie differentiation.

SWI/SNF complexes consist of 12–15 subunits and are the most frequently mutated chromatin regulators in cancer, with at least nine subunits recurrently mutated and in 20% or more of all cancers collectively^10^. SWI/SNF complexes contain an ATPase catalytic core and have been considered to have two sub-families: BAF (defined by ARID1A/B) and PBAF (defined by ARID2, PBRM1, and BRD7)^11^. SMARCB1 was the first SWI/SNF subunit discovered to be mutant in cancer and has been a focus of interest because of the extremely rapid and penetrant cancers that result from its inactivation. It has been of additional interest due to the remarkably unaltered genomes found in SMARCB1-mutant cancers demonstrating potent cancer driving activity caused by SMARCB1 loss. An emerging theme for SWI/SNF tumor suppressor subunits is that their loss, rather than inactivating SWI/SNF complexes, leads to aberrant residual SWI/SNF complexes that are essential for the cancer phenotype. Indeed, there exist several synthetic lethal relationships between paralog subunits of the inactivated tumor suppressor. For example, cancer cell lines mutant for ARID1A are specifically dependent upon its paralog ARID1B whereas cell lines lacking SMRACA4 are specifically dependent upon its paralog SMARCA2^12–14^. Together, these findings have suggested the paradigm that inactivation of SWI/SNF tumor suppressor subunits results in oncogenic activity of residual SWI/SNF complexes that drive cancer cell growth. However, in the case of SMARCB1, the mechanism has been less clear. SMARCB1 has been classified as a core SWI/SNF subunit as it was thought present in all SWI/SNF variants. As is the case with ARID1A and SMARCA4, inactivation of SMARCB1 does not equate to complete inactivation of SWI/SNF complexes as the catalytic ATPase subunit has been shown essential for the survival of RT cells^15^. The mechanism underlying this dependency has been unclear as there are no paralogs of SMARCB1.

To search for vulnerabilities in these cancers, as part of Project Achilles^16^, we carried out a genome-scale CRISPR-Cas9 loss-of-function screen^17^ using a panel of RT cell lines (*n* = 8). We report that BRD9 (Bromodomain-containing protein 9) is a preferential dependency for *SMARCB1*-mutant RT cells lines. Both shRNA-mediated BRD9 knockdown and CRISPR-Cas9-mediated BRD9 knockout specifically impaired proliferation of *SMARCB1*-mutant RT cells. Mechanistically, we identify a unique BRD9-SWI/SNF subcomplex, which is distinct from BAF and PBAF complexes, and entirely lacks SMARCB1. This complex lacks several core subunits, but incorporates the ATPase subunit SMARCA4 (BRG1) and a newly identified subunit GLTSCR1. Although SMARCB1 loss predominantly affects enhancers, BRD9-containing complexes bind to both active promoters and enhancers, where it contributes to gene expression. Loss of BRD9 results in gene expression changes related with apoptosis regulation, translation, and development regulation. Taken together, our results demonstrate that mutation of SMARCB1 in RT results in a specific dependence upon a BRD9-SWI/SNF complex that lacks SMARCB1. BRD9 is essential for the proliferation of SMARCB1-deficient cancer cell lines, suggesting it as a therapeutic target for these lethal cancers.

## Results

### BRD9 is a vulnerability in RT

**Fig. 1:**
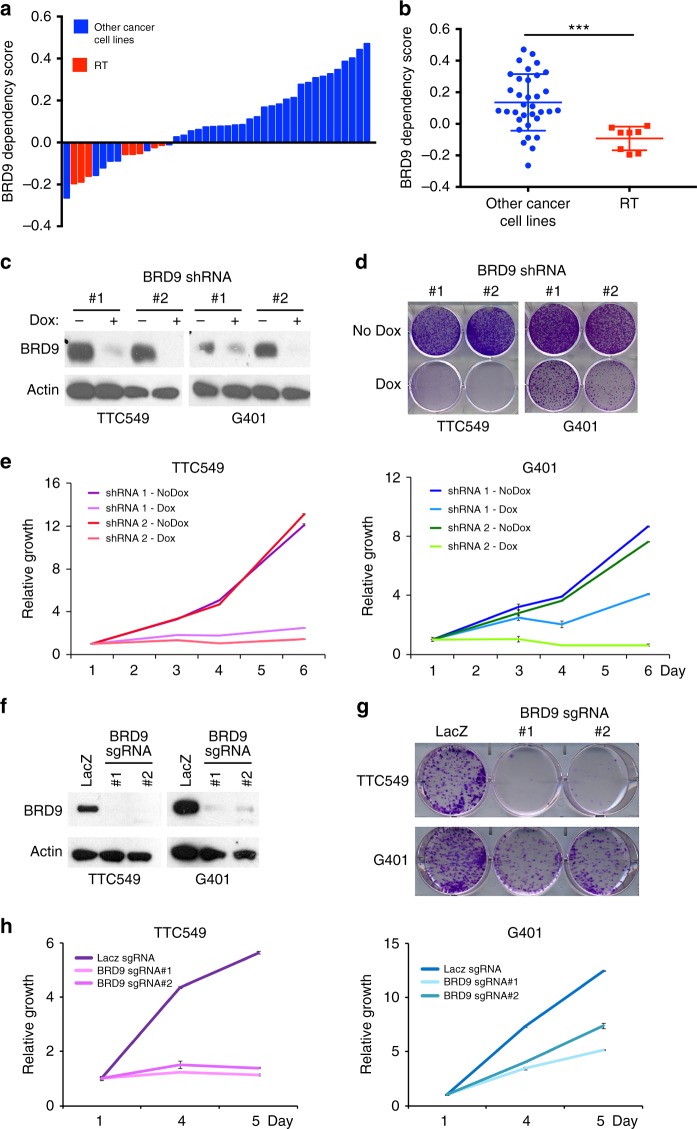
Genome-wide CRISPR-Cas9 screen identified BRD9 as a RT specific dependency. **a**, **b** *SMARCB1*-mutant RT cell lines are more sensitive to BRD9 loss in the CRISPR-Cas9 screen. Red: Rhabdoid Tumor cell lines; Blue: all other cancer cell lines. **c** Western blot showing BRD9 knockdown via shRNA in G401 and TTC549 RT cell lines. **d**, **e** shRNA-mediated BRD9 knockdown impairs cell proliferation: colony forming assay (**d**) and MTT assay (*n* = 3, error bars: SD) (**e**). **f** Western blot showing BRD9 deletion via CRISPR-Cas9 in G401 and TTC549 cell lines. **g**, **h** BRD9 deletion via CRISPR-Cas9 impairs cell proliferation: colony forming assay (**g**) and MTT assay (*n* = 3) (**h**)

We performed a genome-scale CRISPR-Cas9 loss-of-function screen in a panel of eight RT cell lines and 35 other cancer cell lines to identify tumor type-specific vulnerabilities as part of Project Achilles^16,17^. We found that compared to *SMARCB1*-WT cell lines, *SMARCB1*-mutant RT cell lines (*n* = 8) were more sensitive to BRD9 loss (Fig. 1a, b). To validate this finding, we performed shRNA-mediated knockdown of BRD9 expression in RT lines using two independent shRNA hairpins. BRD9 knockdown significantly impaired cell proliferation and viability in TTC549 and G401 RT lines, the latter of which was not included in our initial CRISPR-Cas9 screen (Fig. 1c–e). CRISPR-Cas9 mediated deletion of BRD9 in TTC549, G401, and TTC642 RT lines further substantiated this finding (Fig. 1f–h and Supplementary Fig. 1A). In contrast, BRD9 deletion in *SMARCB1*-WT cancer lines—PANC1 (Pancreatic cancer) and EW8 (Ewing Sarcoma) yielded no significant effect on cell proliferation (Supplementary Fig. 1B). While we were able to efficiently recover BRD9-deficient clones from a control *SMARCB1*-WT cell line (PANC1), we were unable to obtain any BRD9-deficient RT clones in G401 cells (Supplementary Fig. 1C–F), confirming a specific dependency for BRD9 in *SMARCB1*-mutant cells. Together, these results indicate a specific dependency for BRD9 in *SMARCB1*-mutant cells.

### BRD9 defines a unique SWI/SNF subcomplex

**Fig. 2:**
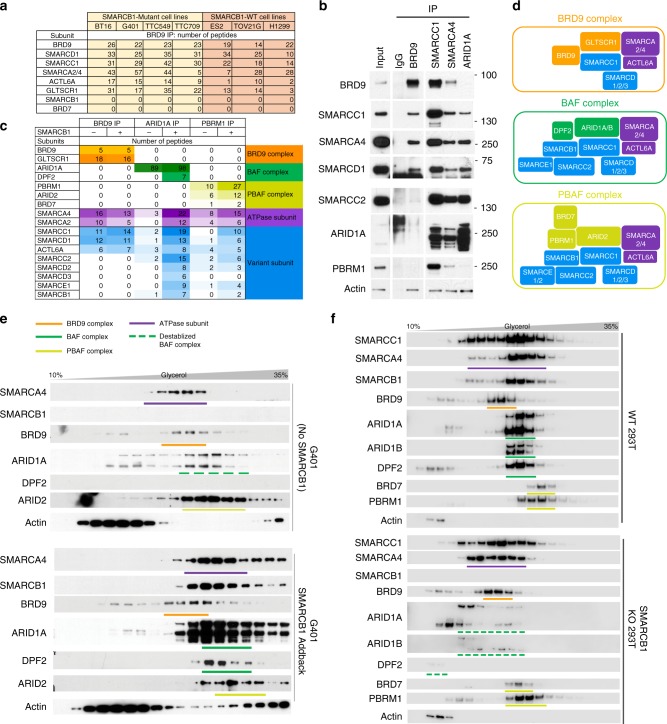
BRD9 defines a unique SWI/SNF subcomplex. **a** Immunoprecipitation-mass spectrometry of BRD9 showing known SWI/SNF subunits from four RT (*SMARCB1*-Mutant) cell lines and three non-RT (*SMARCB1*-WT) cell lines. Source data are provided as a Source Data file. **b** Immunoprecipitation of BRD9 in RT cell line G401 cells and blotted with indicated SWI/SNF subunits. **c** Immunoprecipitation-mass spectrometry of BRD9, ARID1A, PBRM1 in RT cell line G401 without or with SMARCB1 re-expression, showing differential IP enrichment of BRD9, BAF, and PBAF complex subunits. **d** Model of BRD9-SWI/SNF subcomplex in comparison to BAF and PBAF complexes, color for each subunit is consistent with what described in Fig. 2c. **e**, **f** Glycerol gradient of SWI/SNF complexes in G401 cells without (top half) or with SMARCB1 addback (bottom half) (**e**); or in HEK293T parental (top half) or isogenic SMARCB1 KO cells (bottom half) (**f**). BRD9 complexes are slightly smaller than BAF and PBAF complexes and there is a shift of core subunits (SMARCC1, SMARCA4) from BAF complexes to BRD9 complexes in the absence of SMARCB1

BRD9 belongs to the bromodomain superfamily, and it has been shown that it can interact with subunits of the SWI/SNF complex such as SMARCA4^18,19^. However, its role within the SWI/SNF complex has remained largely unclear. To investigate the mechanism by which BRD9 maintains RT cell survival and its relationship with SMARCB1, we purified and identified endogenous BRD9-associated proteins in four different RT cell lines using mass spectrometry. As anticipated, members of the SWI/SNF complex were consistently enriched in BRD9 IP in all four RT cell lines, including SMARCA2/4 (BRM/BRG1), SMARCC1 (BAF155), SMARCD1 (BAF60A), and ACTL6A (BAF53A) (Fig. 2a). However, numerous well-characterized SWI/SNF subunits including SMARCC2 (BAF170), SMARCE1 (BAF57), ARID1A/B (BAF complex specific), ARID2, BRD7, and PBRM1 (PBAF complex specific) were absent. BRD9 IP and subsequent western blot confirmed the mass spectrometry result in G401 (Fig. 2b). To determine if the unique subunit association pattern was caused by the loss of SMARCB1 in RT cells, we performed BRD9 IP-mass spectrometry in G401 RT cells following SMARCB1 re-expression, and found that BRD9 still did not pull down the aforementioned subunits, or, interestingly, even SMARCB1 itself (Fig. 2c). Conversely, while immunoprecipitation of core subunit SMARCC1 (BAF155) or of ATPase subunit SMARCA4 pulled down BRD9 (Fig. 2b), neither ARID1A (specific for BAF complex) nor PBRM1 (specific for PBAF complex) pulled down BRD9 (Fig. 2c). These results indicate that BRD9—while interacting with numerous SWI/SNF subunits—is not part of either BAF or PBAF complexes^11^. To evaluate the composition of BRD9 subcomplex in non-RT cells, BRD9 was immunoprecipitated from three cell lines wild-type for *SMARCB1* (Fig. 2a). In all cases, SWI/SNF subunits co-purified but never subunits specific for either the BAF or PBAF families.

To further exclude the possibility that our finding was an artifact of antibody selectivity (i.e. antibody binding to BRD9 impairs its association with other proteins), we exogenously expressed BRD9 with a C-terminal HA tag in G401 cells and performed HA IP-mass spectrometry. This observation was consistent with endogenous BRD9 purification (Source data file) and indicated that BRD9 is found exclusively in a non-BAF, non-PBAF SWI/SNF subcomplex. Intriguingly, when we systematically compared the mass spectrometry data from all endogenous BRD9 purification as well as the HA-tag IP across all tested cell lines, we noticed that another protein, GLTSCR1 (also known as BICRA, BRD4 Interacting Chromatin-Remodeling Complex Associated Protein), was consistently co-purified at a comparable efficiency to other members such as SMARCA2/4, SMARCC1, but was notably absent from BAF and PBAF complexes (Fig. 2a, b), confirming reports that GLTSCR1 can interact with SWI/SNF complex^20^ and constitutes a distinct SWI/SNF subcomplex (termed GBAF)^21^. These results are summarized in Fig. 2d.

To assess the relationship of BRD9-SWI/SNF subcomplex to the BAF and PBAF complexes, we performed a series of glycerol sedimentation assays in multiple cell lines. In G401 RT cells, we found that BRD9, BAF, and PBAF complexes are modestly separated across gradient fractions in the absence of SMARCB1 and the SMARCA4 ATPase subunit overlapped primarily with fractions containing BRD9 rather than those occupied by BAF or PBAF subunits (Fig. 2e). Upon re-expression of SMARCB1 using our previously established inducible re-expression system, the assembly of BAF complex was noticeably enhanced. Additionally, SMARCA4 shifted towards fractions associated with the BAF complex. This shift of SMARCA4 following SMARCB1 re-expression suggests competition between BRD9 and BAF complex assembly. To further evaluate the relationship between BRD9 and BAF complexes, we conducted a second glycerol sedimentation assay in HEK293T cells and an isogenic derivative knocked out for *SMARCB1* cells. In parental (SMARCB1 intact) cells, BRD9, BAF, and PBAF complexes were well separated across the gradient and peak in distinct fractions (Fig. 2f). While the SMARCA4 ATPase subunit broadly occupied fractions containing BRD9, BAF, and PBAF, it was most concentrated in those associated with BAF. Knockout of SMARCB1 notably altered the gradient. BAF complex subunits ARID1A/B and DPF2 demonstrated reduced levels, and a shift to smaller fractions. Likewise, SMARCA4 shifted from broad occupation of BRD9, BAF, and PBAF-associated fractions to those associated mostly with BRD9. BRD9, however, was minimally affected by the loss of SMARCB1. These changes are reminiscent of assemblies observed in parental G401 cells prior to SMARCB1 re-expression. As further validation, we performed glycerol sedimentation in the HCT116 (*SMARCB1*-WT) colon cancer cell line. The result recapitulated the phenotype demonstrated in G401 *SMARCB1* re-expressed and HEK293T *SMARCB1-*WT cells (Supplementary Fig. 2A). Immuno-depletion assay in G401 cells without and with SMARCB1 re-expression showed that BRD9 specifically depletes SMARCD1 and to a lesser degree SMARCA4, while as predicted it has no effect upon a PBAF specific subunit ARID2. Upon SMARCB1 re-expression the effect of BRD9 immuno-depletion upon SMARCD1 and SMARCA4 is largely diminished (Supplementary Fig. 2B). Together, these experiments indicate that BRD9-containing complexes compete with SMARCB1-containing complexes for incorporation of other SWI/SNF subunits.

### Loss of BRD9 disrupts the BRD9-SWI/SNF subcomplex

**Fig. 3:**
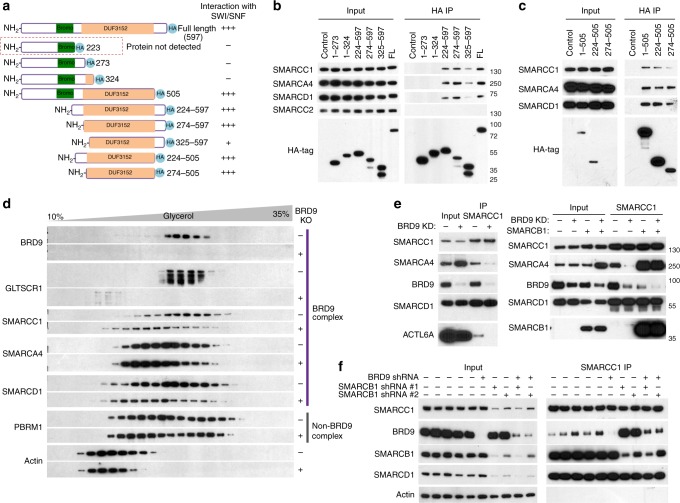
Loss of BRD9 disrupts BRD9-SWI/SNF complex. **a** Illustration of BRD9 domains and deletion mutants and a summary of BRD9 deletion mutants with SWI/SNF complex. – indicates no interaction; +++indicates strong interaction; +indicates weak interaction. **b**, **c** Immunoprecipitation of HA-tagged BRD9 truncation mutants and western blot showing their interactions with SWI/SNF subunits. The DUF3512 domain is essential for BRD9 interaction with SWI/SNF complex subunits. **d** Glycerol gradient of G401 cells with control CRISPR-Cas9 (−) or BRD9 targeting CRISPR-Cas9 (+). Loss of BRD9 leads to GLTSCR1 eviction and substantial degradation. **e** Western blot of input and SMARCC1 IP from G401 RT cells without or with BRD9 knockdown and without or with SMARC1B addback. Loss of BRD9 impairs SMARCA4 and SMARCC1 interaction only in a *SMARCB1*-deficient context. **f** Western blot of input and SMARCC1 IP from H1299 cells with BRD9 knockdown, SMARC1B knockdown or both. Loss of SMARCB1 leads to increased BRD9-SMARCC1 interaction

To investigate the mechanism by which BRD9 is preferentially essential in SMARCB1 cells, we examined the role of BRD9 in complex assembly. BRD9 contains a bromodomain and an uncharacterized DUF3512 domain^19^. To dissect the functions of these two domains, we made several truncation mutants (Fig. 3a). By performing IP with an anti-HA antibody and immunoblotting, we found that the DUF3512 domain is both sufficient and necessary for BRD9 interactions with other subcomplex subunits while the bromodomain is dispensable (Fig. 3b, c), demonstrating that DUF3512 functions as a scaffolding domain essential for interaction with the tested SWI/SNF subunits. Consistent with this finding, while BRD9 and BRD7 share a highly similar bromodomain, their DUF3512 domains are quite distinct, explaining why BRD7 and BRD9 are recruited to different complexes (Supplementary Fig. 3).

We then evaluated the effect of BRD9 loss on the complex integrity in *SMARCB1*-mutant cells using glycerol sedimentation assays. Strikingly, knockout of BRD9 via CRISPR/Cas9 editing in G401 cells led to complete eviction of GLTSCR1 from the subcomplex (Fig. 3d), while other subunits in the complex (SMARCC1 and SMARCA4) were moderately affected, with reduced level in BRD9 fractions. This result also indicates that BRD9 is essential for incorporation of GLTSCR1 to the complex. To further examine the consequences of BRD9 loss upon complex integrity, we next performed IP of core SWI/SNF subunit SMARCC1. While association of SMARCC1 with SMARCD1 was unaffected by BRD9 deletion, association with both SMARCA4 and ACTL6A were markedly impaired indicating that loss of BRD9 in RT cells results in near complete disassociation of the core SMARCC1 from the ATPase subunit.

To evaluate the contribution of SMARCB1 absence to this profound disassociation, we re-expressed SMARCB1 in RT cell lines and again evaluated the consequences of BRD9 deletion. The presence of SMARCB1 fully rescued the association of SMARCC1 with SMARCA4 and ACTL6A in the absence of BRD9 (Fig. 3e), suggesting BRD9 is essential for formation of functional SWI/SNF complexes, in the absence of SMARCB1-containing complexes. To further evaluate this, we knocked-down SMARCB1 in a non-RT cell line (H1299) and performed a SMARCC1 IP. Upon SMARCB1 loss the interaction between BRD9 and SMARCC1 was markedly increased (Fig. 3f). Conversely, knockdown of BRD9 had no effect upon SMARCC1 association with SMARCB1, suggesting that SMARCB1-containing complexes are either more abundant or that SMARCB1 protein is limiting in H1299 cells. This is also consistent with glycerol gradient data that BAF complex is dominant in SMARCB1 wild-type cells. Taken together, our data suggest that there is an intrinsic competition between the assembly of SMARCB1-containing complex and BRD9-containing complex, and that SMARCB1 could potentially bind to SMARCC1 with a higher affinity. Following this logic, in RT cells, upon SMARCB1 mutation, the assembly of BRD9-SWI/SNF subcomplex is enhanced and becomes essential.

### Genome-wide binding of BRD9 and transcriptional regulation

**Fig. 4:**
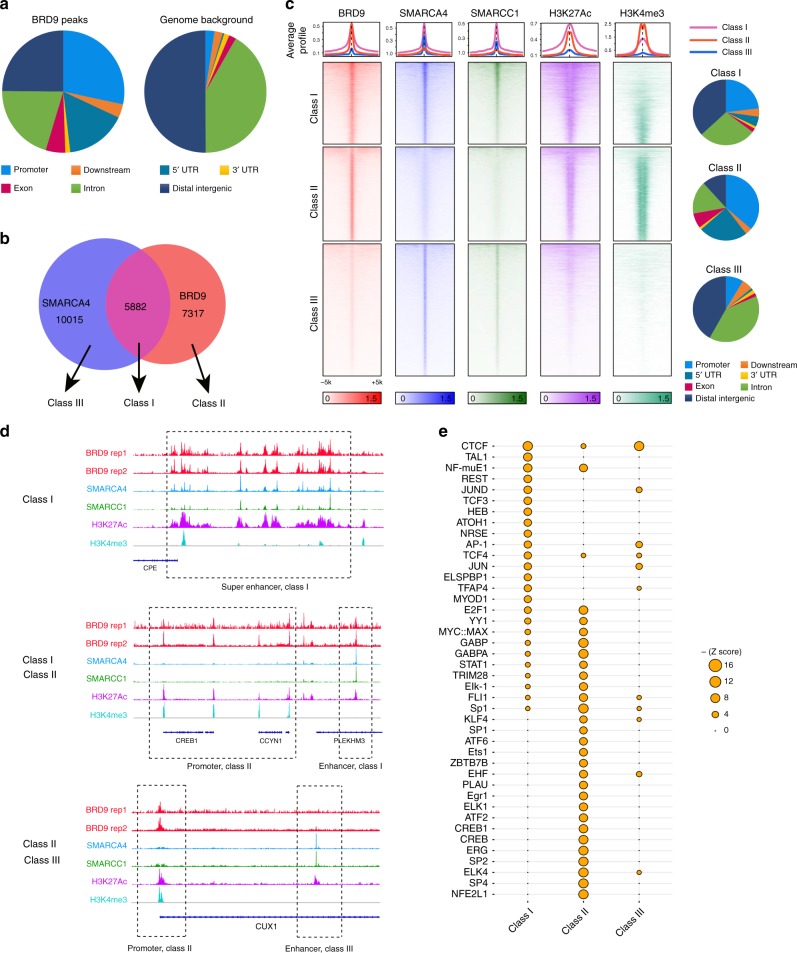
Genome-wide targeting of BRD9 directly regulates gene expressions. **a** The genomic distribution of BRD9 binding sites in G401 cells. **b** Venn diagram of overlap between SMARCA4 and BRD9 peaks and definition of the three classes of BRD9 peaks. **c** The average ChIP enrichment profile of BRD9, SMARCA4, SMARCC1, H3K27Ac, and H3K4me3 over class I, class II, and class III peak regions (top). Heatmap illustrating the ChIP-seq signal 5 kb up- and downstream of the different peak classes. The genomic distribution of each class is shown in the right. **d** Examples of BRD9, SMARCA4, SMARCC1, H3K27Ac, and H3K4me3 ChIP-seq at representative Class I, II, and III peak regions. **e** Comparison of motif enrichment in different peak regions. Size of the circle represents the significance of the motif

SMARCB1 had previously been considered a core SWI/SNF subunit, but our results indicated that BRD9-containing complexes lack SMARCB1. We thus sought to evaluate the targeting of the BRD9 subcomplex to chromatin. As endogenous BRD9 ChIP-seq was not successful in our hands and others^19^, we took the strategy of CETCh-seq (CRISPR epitope tagging ChIP-seq)^22^ to add an HA-tag to the endogenous locus of BRD9 at the C-terminus and perform HA-tag ChIP-seq. Of note, adding an HA tag did not impair the formation of BRD9-SWI/SNF subcomplex (Supplementary Fig. 4). Analysis of the ChIP-seq data of BRD9, SMARCA4, SMARCC1, as well as histone modifications (H3K27Ac, H3K4Me3)^7^ revealed that: (1) binding of BRD9 was enriched in promoters and 5′ UTRs (Fig. 4a); (2) nearly half of BRD9 peaks overlap with SMARCA4 peaks (Class I peaks, Fig. 4b), and the overlapping peaks often co-localize at both enhancers and active promoters (top half of the Class I peaks have H3K27Ac; the bottom have both H3K27Ac and H3K4me3 in Fig. 4c; examples are shown in Fig. 4d); (3) the other half of BRD9 peaks (Class II peaks, which have little or no SMARCA4/SMARCC1 binding) are enriched mostly in promoters (Fig. 4c, d). This raises the possibility that BRD9 might function as free form or other smaller subcomplexes, which is consistent with glycerol gradient experiments from our data (Fig. 2c, d) and others^23^; (4) SMARCA4 binding sites that overlap with no or little BRD9 (Class III peaks) showed enhancer-like features, indicating the binding of BAF/PBAF complexes (Fig. 4c, d); and (5) Class I peaks are wider than Class II and III (Supplementary Fig. 5). Collectively these results demonstrate that BRD9, while sharing some similarities with other SWI/SNF complexes also has distinct binding patterns including a notable enrichment at promoters.

To evaluate whether the distinct classes of BRD9 binding sites may have different functions with respect to transcriptional regulation, we next analyzed correlation with transcription factor binding motifs that are enriched in these three categories of binding sites (Fig. 4e). Consistent with our previous findings of motifs enriched at SMARCA4 binding sites, the shared regions between BRD9 and SMARCA4 were enriched with AP-1 and CTCF motifs^8^. While there was substantial overlap in gene sets associated with Class I and Class II sites, class II regions revealed more unique motifs, including (1) ETS family of transcription factors such as ETS1, ERG, and FLI1. A recent report that the SWI/SNF complex interacts with EWS-FLI1 and targets tumor-specific enhancers in Ewing sarcomas^24^, suggesting a broader relationship between SWI/SNF complexes with ETS family transcription factors; (2) ELK1, a target of the Ras/Raf/MAPK pathway, (3) ATF2 and CREB, related with the cAMP and apoptosis pathways, which have been shown to interact with SWI/SNF^25^. This suggests that BRD9 may regulate multiple pathways, likely via interacting with various transcription factors.

**Fig. 5:**
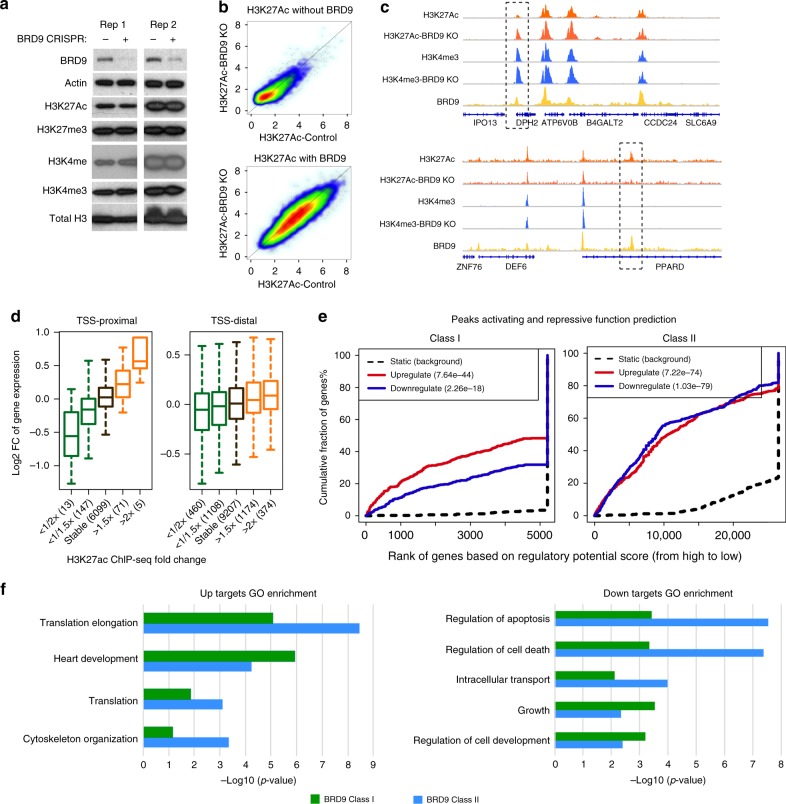
BRD9 directly regulates gene expression. **a** Western blot of selected histone modifications in G401 cells with or without BRD9. **b**, **c** Scatterplot of the average H3K27Ac (**b**) and H3K4me3 (**c**) ChIP-seq signals in *H3K27Ac*-WT and *H3K27Ac*-KD BRD9 over BRD9 binding sites. Loss of BRD9 leads to bidirectional changes of H3K27Ac and H3K4me3. **d** Correlation between the closest gene expression changes with H3K27ac signal changes at TSS-proximal and TSS-distal regions upon BRD9 deletion. In the box plots, bounds of the box spans from 25 to 75% percentile, the center line represents median, and whiskers visualize 5 and 95% of the data points. **e** BETA activating/repressive function prediction of the BRD9 class I and class II peaks in G401 cell. The red and the purple lines represent BRD9 upregulated and downregulated genes, respectively. The dashed line indicates the non-differentially expressed genes as background. Genes are cumulated by the rank on the basis of the regulatory potential score from high to low. The regulatory potential in the *x*-axis represents the likelihood of a gene being regulated by BRD9, calculated by considering both the number of BRD9 binding sites and the distance between gene TSS and the BRD9 peak (see details from methods). *P*- values that represent the significance of the upregulated or downregulated gene group distributions are compared with the static gene group by the Kolmogorov–Smirnov test. **f** GO terms enrichment of BRD9 class I and BRD9 class II direct up (left) and down (right) target genes upon BRD9 deletion

We have previously shown that SMARCB1 (present in BAF and PBAF complexes), SMARCA4 (BAF, PBAF, and BRD9 complexes), and ARID1A (BAF complexes only) serve important roles in the establishment of active chromatin at enhancers such that their loss results in marked reduction of active histone marks. To investigate contributions of BRD9, we performed western blot against enhancer and promoter-associated histone modifications (Fig. 5a) in G401 cells. Unlike loss of other members such as SMARCB1, SMARCA4, and ARID1A^7,8,26^, loss of BRD9 did not change the global levels of key histone marks such as H3K27Ac and H3K4me3 (Fig. 5a), although the presence of BRD9 was positively correlated with H3K27Ac and H3K4me3 signal (Fig. 5b, Supplementary Fig. 6A). However, analysis of ChIP-seq profiles for H3K27Ac and H3K4me3 in G401 BRD9-KO and BRD9-WT cell showed both increases and decreases at promoters and enhancers (Fig. 5b, c, Supplementary Fig. 6), and changes of H3K27Ac at these loci were positively correlated with gene expression changes in both TSS-proximal and TSS-distal regions (Fig. 5d). To directly evaluate the role of BRD9 in transcriptional regulation, we used our published algorithm BETA (Binding and Expression Target Analysis)^27^ to: (1) examine whether BRD9 has activating or repressive, or both, function in regulating downstream gene expression; (2) identify BRD9 direct target genes as those with both BRD9 binding and demonstrating BRD9-dependent gene expression changes. In using BETA, we found BRD9 binding was enriched at both activated and repressed genes rather than the static genes (Fig. 5e). This demonstrates that BRD9 function is required for both activating and repressive functions in regulating gene expression. Given the distinct biochemical assemblies and binding of BRD9 in class I and class II sites we next analyzed these genes independently. We utilized GO (Gene Ontology) analyses to determine whether these direct target genes were enriched for previously identified functional categories. While there was substantial overlap in gene sets associated with Class I sites and Class II sites, there was stronger association of Class II sites with GO terms related to apoptosis and cell death while class I sites were preferentially associated with development (Fig. 5f), thus raising the possibility of distinct functional roles for BRD9 when bound with and without canonical SWI/SNF subunits SMARCA4 and SMARCC1.

To further look at primary and secondary effects of BRD9 loss on transcriptional changes, we next performed a BRD9 deletion time-course (Day 1, 2, 3, and 5) RNA-seq experiment (Supplementary Fig. 7A, B). Comparison of the distance of BRD9 binding to differentially expressed genes and static genes revealed that BRD9 was bound closer to differentially expressed genes (both activated and repressed) than to unchanged genes suggesting direct contribution of BRD9 to both activation and repression (Supplementary Fig. 7C). Analyses revealed that that transcriptional changes caused by BRD9 loss occurred in a time-dependent manner. For example, early on at Days 1 and 2 genes sets associated with chromatin structure are enriched, such as nucleosome assembly and gene silencing. Later, at days 3–5, gene sets associated with downstream functions such as differentiation, development and apoptosis are enriched (Supplementary Fig. 7D, E).

## Discussion

Taken together, our work here identifies BRD9 as a preferential dependency for *SMARCB1*-mutant RT cells. In understanding the mechanism, we report a BRD9-containing SWI/SNF subcomplex (GBAF or ncBAF), which is distinct from originally identified BAF and PBAF subcomplexes. In characterizing the assembly of this BRD9 SWI/SNF subcomplex, we identified an intrinsic competition between BRD9 and SMARCB1 in forming SWI/SNF complexes such that loss of SMARCB1, as occurs in RT, results in enhanced formation of residual BRD9-containing complexes. Mechanistically, it is disruption of the SMARCB1-BRD9 balance that results in the specific dependence upon BRD9 in RT.

We have previously shown that SWI/SNF complexes are enriched at active enhancers, and are directly involved in regulating enhancer activity. In this current study, we found that while BRD9 co-localizes with SMARCA4/SMARCC1 (Class II sites) at active enhancers, BRD9 also binds to regions that lack of SMARCA4/SMARCC1 (Class I sites), which are mostly at active promoters. By integrating BRD9 ChIP-seq and BRD9 deletion RNA-seq data, we found that BRD9 has both activating and repressing roles in regulating gene expressions, both at promoters and enhancers.

Our findings suggest BRD9 as a therapeutic target in RT. Relevant to this, a BRD9 bromodomain-specific inhibitor was earlier developed and shown to have efficacy in inhibiting leukemia cell proliferation^19^. However, we found that the bromodomain of BRD9 is dispensable for its role in maintaining the integrity of residual SWI/SNF complexes in RT. Instead we found that the previously uncharacterized DUF3512 domain is essential in this role. Consistent with this, we found that RT cells were insensitive to the BRD9 bromodomain inhibitors (Supplementary Fig. 8) suggesting that degradation of BRD9 would instead be required to have therapeutic benefit. As a BRD9 degradation molecule was recently reported^28,29^, it will be of great interest to test its efficacy in RT cancers.

## Methods

### Cell culture

G401, H1299, HCT116, and ES-2 cell lines were purchased from American Type Culture Collection (ATCC). BT16, TOV21G, TTC642, TTC709, and TTC549 cells were maintained in the lab. G401 and BT16 cells were cultured in DMEM with 10% FBS; ES-2 cells were cultured in McCoy’s with 10% FBS; HCT116, TTC549, TTC709, TTC642 cells were cultured in RPMI with 10% FBS. KP-MRT-RY was a kind gift from Yasumichi Kuwahara at the Kyoto Prefectural University of Medicine. Cell lines were Mycoplasma negative, and identities were validated by SNP fingerprinting to 96 SNPs by Fluidigm at the Broad Genomics Platform as described previously^30^. SMARCB1 inducible re-expression stable G401 cell line was maintained in media with Tet-System Approved FBS (CloneTech, Cat. #631106). To induce SMARCB1 re-expression, cells were treated with doxycycline (1 μg/ml, EMD Millipore) for indicated time. For shRNA-mediated knockdown, cells were transduced with lentiviral shRNAs and selected with puromycin for 72 h before seeding for proliferation or colony formation assays. Proliferation assays were conducted with either an MTT Cell Proliferation Kit (Roche Diagnostics, Cat. #11465007001) or CellTiter-Glo 2.0 (Promega, Cat. # G9242). Colony formation assays were conducted by staining cells for 20 min with crystal violet staining solution (0.05% crystal violet, 1% formaldehyde, 1% PBS, 1% methanol). shRNAs were obtained from the RNA interference (RNAi) screening core facility at the Dana-Farber Cancer Institute and cells were lentivirally infected and selected under puromycin for 72 h before seeding for proliferation assay. Cell lines were infected with lentiCas9-Blast and stable cell lines expressing Cas9 were selected and maintained under blasticidin; sgRNAs were cloned into lentiGuide-Puro, cells were infected and selected with puromycin. Cell proliferation assays were performed using Cell Proliferation Kit I—MTT (Roche, Cat. #11465007001) or CellTiterGlo 2.0 (Promega, Cat. #G9242).

CRISPR-Cas9 KO construct (SMARCB1) was purchased from Santa Cruz Biotechnology to make 293 T SMARCB1 knockout isogenic cell line.

### CRISPR-Cas9 Screen

Broad Institute’s GeCKOv2 library screen of 43 cancer cell lines, which included 8 RT lines were used for analysis. This library contained ~123,411 sgRNAs with an average of six sgRNAs per gene and 1000 negative control sgRNAs. CRISPR data from Project Achilles used in this manuscript can be downloaded from the Figshare repository (https://figshare.com/s/95d022c461d6d8e45670). These data contain gene dependency scores estimated for each gene and cell line using the CERES algorithm^29^.

### Co-immunoprecipitation and mass spectrometry

Nuclear extracts for co-immunoprecipitation were prepared using the NE-PER Nuclear and Cytoplasmic Extraction Kit (ThermoFisher Scientific, Cat. #78835). Nuclear extracts were diluted to bring down the salt concentration to 150 mM (with protease inhibitor cocktails, Roche). Each IP was rotated with antibodies overnight at 4 °C. Protein G Dynabeads (Life Technologies, Cat. #10009D) were added and rotated at 4 °C for 3 h. Beads were then washed three times with RIPA buffer and resuspended in reducing SDS gel loading buffer. Antibodies to the following proteins were used in the immunoprecipitation and immunoblots: BRD9 (Bethyl Laboratories: A303–781A; 1:3000 for WB); SMARCC1/BAF155 (Santa Cruz: sc9746; 1:3000 for WB); ARID1A (Santa Cruz: sc-32761 for immunoprecipitation; Cell Signaling Technology: 12354 for immunoblotting; 1:1000 for WB); SMARCA4/BRG1 (Santa Cruz: sc17796; 1:500 for WB); GLTSCR1 (Santa Cruz: sc-515086; 1:200 for WB); SMARCC2/BAF170 (Bethyl Laboratories: A301–039A; 1:3000 for WB); SMARCD1/BAF60A (Bethyl Laboratories: A301–595A; 1:3000 for WB); SMARCE1/ BAF57 (Bethyl Laboratories: A300–810A; 1:3000 for WB); SMARCB1/SNF5 (Bethyl Laboratories: A301–087A; 1:5000 for WB); ACTL6A/BAF53A (Bethyl Laboratories: A301–391A; 1:3000 for WB); PBRM1 (Bethyl Laboratories: A301–591A; 1:3000 for WB); HA-tag (Cell Signaling Technology, 3724; 1:3000 for WB); and ACTIN (Cell Signaling Technology: 5125; 1:3000 for WB). For mass spectrometry, equal amounts of nuclear extract were used for each IP. Samples after IP were separated on a NUPAGE 12% Bis-Tris gel, and stained with SimplyBlue SafeStain (Life Technologies). Per IP sample, the whole lane was cut and sent for protein identification at the Taplin Mass Spectrometry Facility at Harvard Medical School.

### Glycerol sedimentation assay

Briefly, cells were harvested at indicated time points, then lysed and homogenized in Buffer A (10 mM HEPES (pH 7.6), 25 mM KCl, 1 mM EDTA, 10% glycerol, 1 mM DTT, and protease inhibitors (complete tablets, Roche) supplemented with 1 mM PMSF) on ice. Nuclei were sedimented by centrifugation (1000× *g* for 10 min), resuspended in Buffer B (10 mM HEPES (pH 7.6), 3 mM MgCl2, 100 mM KCl, 0.1 mM EDTA, 10% glycerol, 1 mM DTT, and protease inhibitors), and further lysed by the addition of ammonium sulfate to a final concentration of 0.3 M. Soluble nuclear proteins were separated by the insoluble chromatin fraction by ultracentrifugation (100,000 × *g* for 20 min) and precipitated with 0.3 g/ml ammonium sulfate for 20 min on ice. Protein precipitate was isolated by ultracentrifugation (100,000 × *g* for 30 min) and resuspended in Buffer A without glycerol. 1 mg of nuclear extract was carefully overlaid onto a 12-ml 10–35% glycerol gradient prepared in a 14-ml 14 × 95 mm polyallomer centrifuge tube (Beckman Coulter, Cat. #331374). Tubes were placed in an SW-40 Ti swing bucket rotor and centrifuged at 4 °C for 16 h at 40,000 × r.p.m. Fractions (~0.6 ml) were collected and used in gel electrophoresis and subsequent western blotting analyses.

### BRD9 inhibitor treatment

Cells were plated and screened in 384-well format as described previously^30^. Briefly, cells were manually plated in white, opaque tissue-culture-treated plates (Corning) at 1000 cells/well. Compounds were tested over a 14-point concentration range (twofold dilution) in duplicate. Compounds were added (1:300 dilution) using a CyBi-Well Vario pin-transfer machine 24 h after plating, and sensitivity was measured using CellTiterGlo (Promega) 72 h after the addition of small molecules. BRD9 inhibitors were kindly provided by Manfred Koeg from Boehringer Ingelheim.

### ChIP-seq experiment

Cells were incubated with 1% formaldehyde for 10 min and subsequently quenched with glycine for 5 min. Cells were washed with PBS three times prior to nuclear extraction. Chromatin was fragmented the adaptive focused acoustics (AFA) technology developed by Covaris for the cell lines. Solubilized chromatin was immunoprecipitated with antibodies against HA-tag (Cell Signaling Technology: 3724; 10 µL per ChIP). Antibody-chromatin complexes were pulled down with Protein G-Dynabeads (Life Technologies), washed, and then eluted. After crosslinking reversal, RNase A, and proteinase K treatment, ChIP DNA was extracted with the Min-Elute PCR purification kit (Qiagen). ChIP DNA was quantified with Qubit dsDNA Assay Kit (Life Technologies). A volume of 5 ng of ChIP DNA per sample was used to prepare sequencing libraries using NEBNext Ultra II RNA Library Prep Kit (New England Biolabs, E7645), and were sequenced with the Nextseq Illumina genome analyzer. Two biological replicates of HA-BRD9 ChIP-Seq were performed in G401 cells.

### Public ChIP-seq data sets

SMARCA4, SMARCC1, H3K27Ac, and H3K4me3 ChIP-seq data from G401 cell were obtained from GSM1835876, GSM1835877, GSM1835878, GSM1835879, GSM1835880, respectively^10^.

### ChIP-seq data analysis pipeline

ChIP-seq data sets were aligned to human genome hg19 using Bowtie^31^ with –m 1–best. We used SPP package^32^ in R to identify the ChIP-seq enriched regions. Parameters window.size = 500, z.thr = 4, and matching input data for each sample were used while peak calling. Peaks genomic distribution was calculated via CEAS software (X. Shirley Liu laboratory), and the sequencing depth normalized ChIP signal was visualized via IGV browser tracks (Fig. 4d, Supplementary Fig. 5).

### Motif analysis

MDSeqPos (X. Shirley Liu laboratory) was applied to identify binding motifs based on BRD9 and BRG1 class I, class II, and class III peaks. All binding sites were trimmed or extended to 600 bp and centered at the center of the peak regions. Motifs with *z*-score < = −10 in at least one class were collected, the enrichment significance in each class were shown in Fig. 4e. Values shown in Fig. 4e are negative z-score output from MDSeqPos, the higher the value, the more significant the motif enriched, value = 0 represents the lack of enrichment.

### RNA-seq experiment

For RNA-Seq, total RNA was extracted using Trizol reagent (Invitrogen) and further purified using RNeasy MinElute Cleanup Kit (Qiagen). A volume of 1 μg of total RNA was used to make the RNA-Seq library using NEBNext Ultra RNA Library Prep Kit (New England Biolabs, E7530) and sequenced with the NextSeq Illumina genome analyzer. Each sample has two biological replicates.

### RNA-seq data analysis

RNA-seq data sets were aligned to human genome hg19 using STAR^33^ with ENCODE standard options. RSEM^34^ was used to do the transcript quantification, and differential expression analysis were performed with DESeq2^35^.

### Selected peaks activating and repressive function analysis

We used binding and expression target analysis pipeline^27^ with parameter–da 500 to predict different classes of peaks’ activating and repressive function. Regulatory potential for each gene was calculated as $S_g = \mathop {\sum }\nolimits_{i = 1}^k e^{ - (0.5 + 4\Delta _i)}$. All peaks (*k*) near the transcription start site (TSS) of the gene (*g*) within a 100 kb are considered. ∆ is the exact distance between a binding site and the TSS proportional to 100 kb (∆ = 0.1 means the exact distance = 10 kb). *P*-values listed in the top left were calculated by Kolmogorov–Smirnov test to measure the significance of the upregulated genes group or downregulated genes group relative to static genes group.

### Gene ontology analysis

BRD9 target genes that predicted by BETA in class I and class II were used to do the gene ontology (GO) analysis. DAVID^36^ was performed to do the GO enrichment. GO terms with *P*-value <= 1e-3 in either class I target genes or class II target genes were collected and shown in Fig. 5f.

### Reporting summary

Further information on research design is available in the Nature Research Reporting Summary linked to this article.

## Supplementary information

Supplementary Information

Peer Review File

Reporting Summary

Source Data

## Data Availability

A reporting summary for this Article is available as a Supplementary Information file. RNA-seq and ChIP-seq data that support the findings of this study have been deposited in the Gene Expression Omnibus (GEO) under accession code GSE120235. The complete mass spectrometry files for BRD9-interacting proteins are provided in a Source Data file. The source data underlying Figs. 2E, 2F, 3B, 3C, 3D, and 3E are provided in Supplementary Information. All other data are available from the corresponding author upon reasonable request.
