# Author Correction: BRD9 defines a SWI/SNF sub-complex and constitutes a specific vulnerability in malignant rhabdoid tumors

**DOI:** 10.1038/s41467-019-12524-8

**Published:** 2019-09-26

**Authors:** Xiaofeng Wang, Su Wang, Emma C. Troisi, Thomas P. Howard, Jeffrey R. Haswell, Bennett K. Wolf, William H. Hawk, Pilar Ramos, Elaine M. Oberlick, Evgeni P. Tzvetkov, Aaron Ross, Francisca Vazquez, William C. Hahn, Peter J. Park, Charles W. M. Roberts

**Affiliations:** 1 Department of Molecular and Systems Biology, Geisel School of Medicine, Dartmouth College, Hanover, NH 03756 USA; 2 Department of Pediatric Oncology, Dana-Farber Cancer Institute, Boston, MA 02215 USA; 3 Department of Biomedical Informatics, Harvard Medical School, Boston, MA 02115 USA; 4 Broad Institute of Harvard and MIT, 415 Main Street, Cambridge, MA 02142 USA; 5 Comprehensive Cancer Center and Department of Oncology, St. Jude Children’s Research Hospital, Memphis, TN 38105 USA; 6 Department of Medical Oncology, Dana-Farber Cancer Institute, Boston, MA 02215 USA

**Keywords:** Biochemistry, Cancer, Epigenetics

Correction to: *Nature Communications* 10.1038/s41467-019-09891-7, published online 23 April 2019.

The original version of this Article omitted the eleventh author Aaron Ross, who is from the ‘Comprehensive Cancer Center and Department of Oncology, St. Jude Children’s Research Hospital, Memphis, TN 38105, USA’. Consequently, the following was added to the Author contributions: ‘A.R. performed experiments’. This has been corrected in both the PDF and HTML versions of the Article.

