# Supplementary material for: BRD9 defines a SWI/SNF sub-complex and constitutes a specific vulnerability in malignant rhabdoid tumors
Source: Nat Commun. 2019 Apr 23;10:1881. doi: 10.1038/s41467-019-09891-7 (PMC6479050; doi:10.1038/s41467-019-09891-7)
Supplement: Supplementary file 1 — Supplementary Information [file 41467_2019_9891_MOESM1_ESM.pdf]

BRD9 defines a novel SWI/SNF sub-complex and constitutes a specific vulnerability in malignant rhabdoid tumors

Wang et al.

Supplementary Information

Supplementary Figure 1

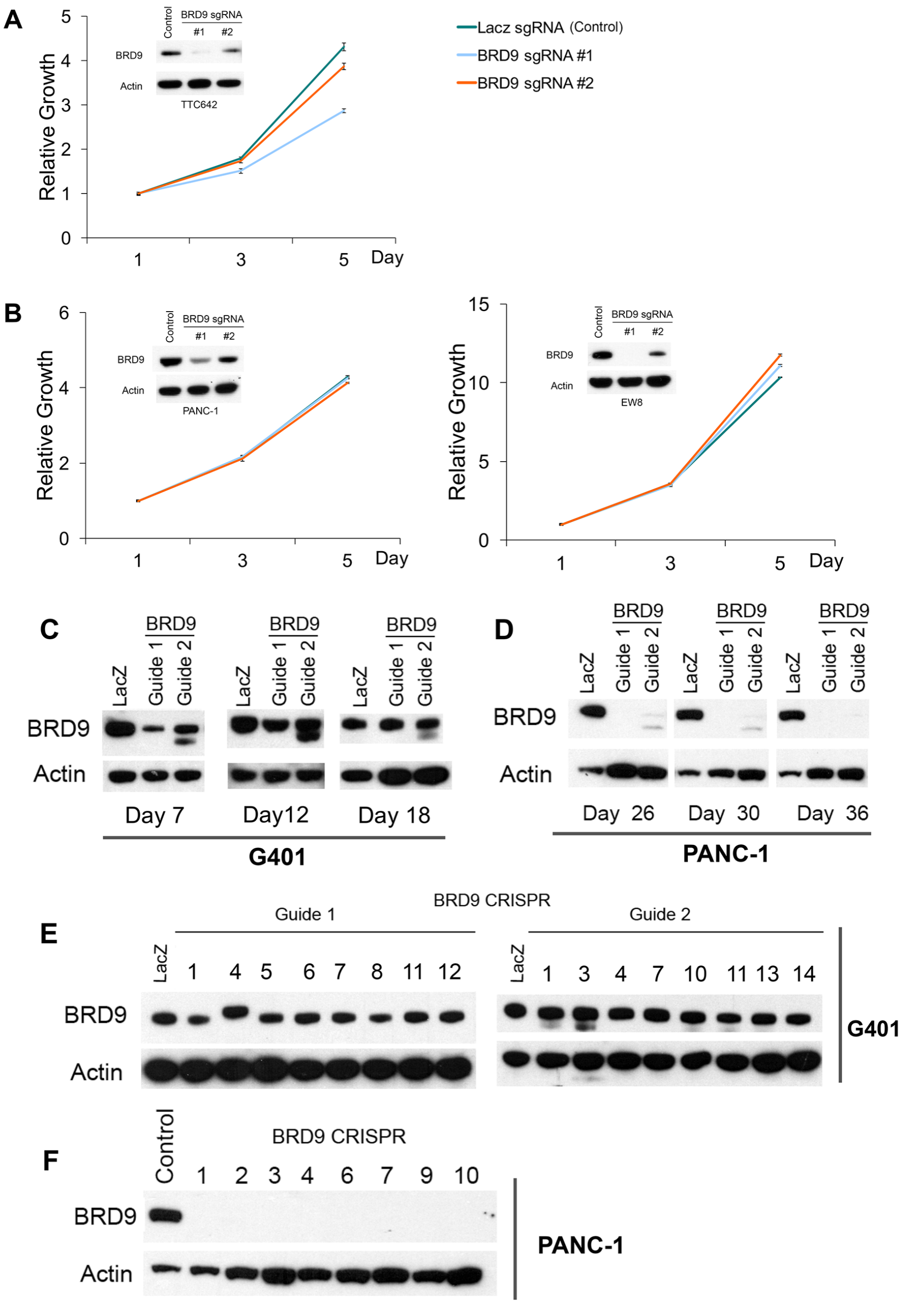

Supplementary Figure 1

A. BRD deletion significantly impairs MRT cell line TTC642 proliferation (*n=3*, *error bars: SD)*

B. BRD9 deletion has no effect on PANC-1 and EW8 cell proliferation (*n=3*);

C. Western blot showing rapid negative selection of BRD9 wildtype (unedited) G401 cells;

D. Western blot showing long-lasting BRD9 knock-out (edited) PANC-1 cells;

E. Western blot showing all singe cell clones recovered from G401 cells were BRD9 wild-type (*n=16*);

F. Western blot showing all singe cell clones recovered from PANC-1 cells were BRD9 knock-out (*n=8*).

Supplementary Figure 2

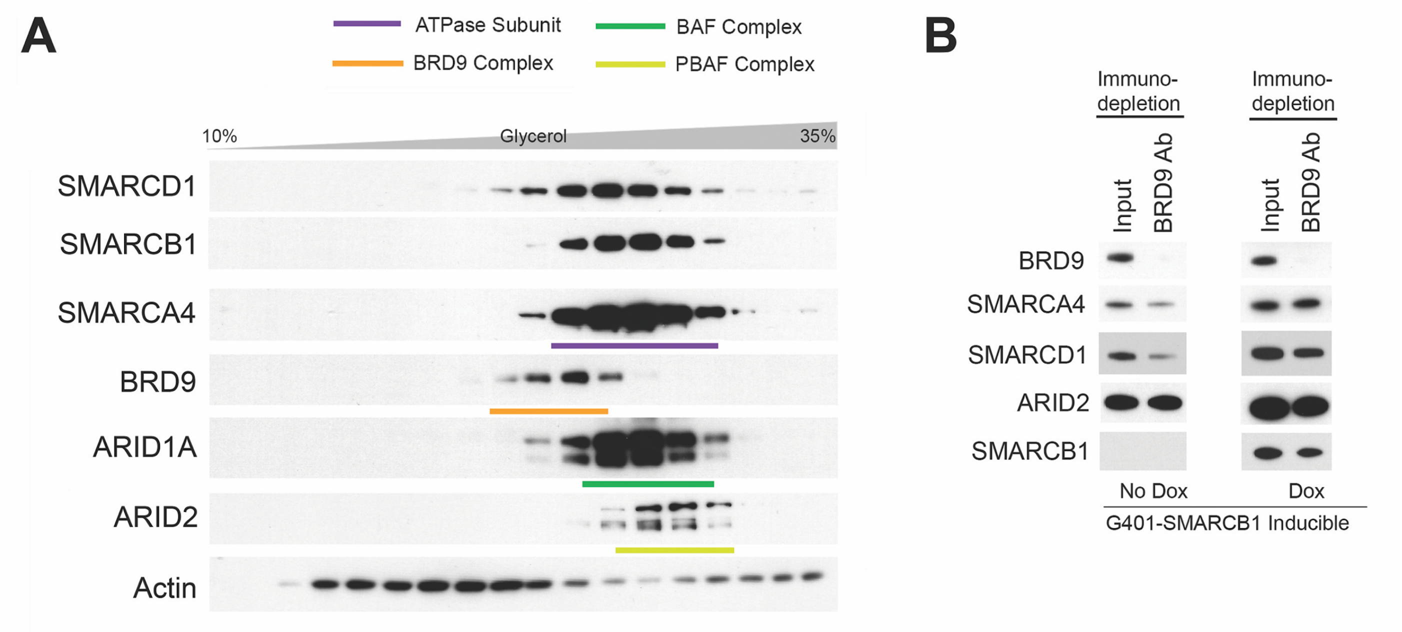

Supplementary Figure 2

A. Glycerol gradient of HCT116 (*SMARCB1*-WT) colon cancer cell line showing BRD9, BAF and PBAF complex subunits.
B. Immuno-deletion of SWI/SNF subunits using BRD9 in G401 cells without or with SMARCB1 addback

Supplementary Figure 3

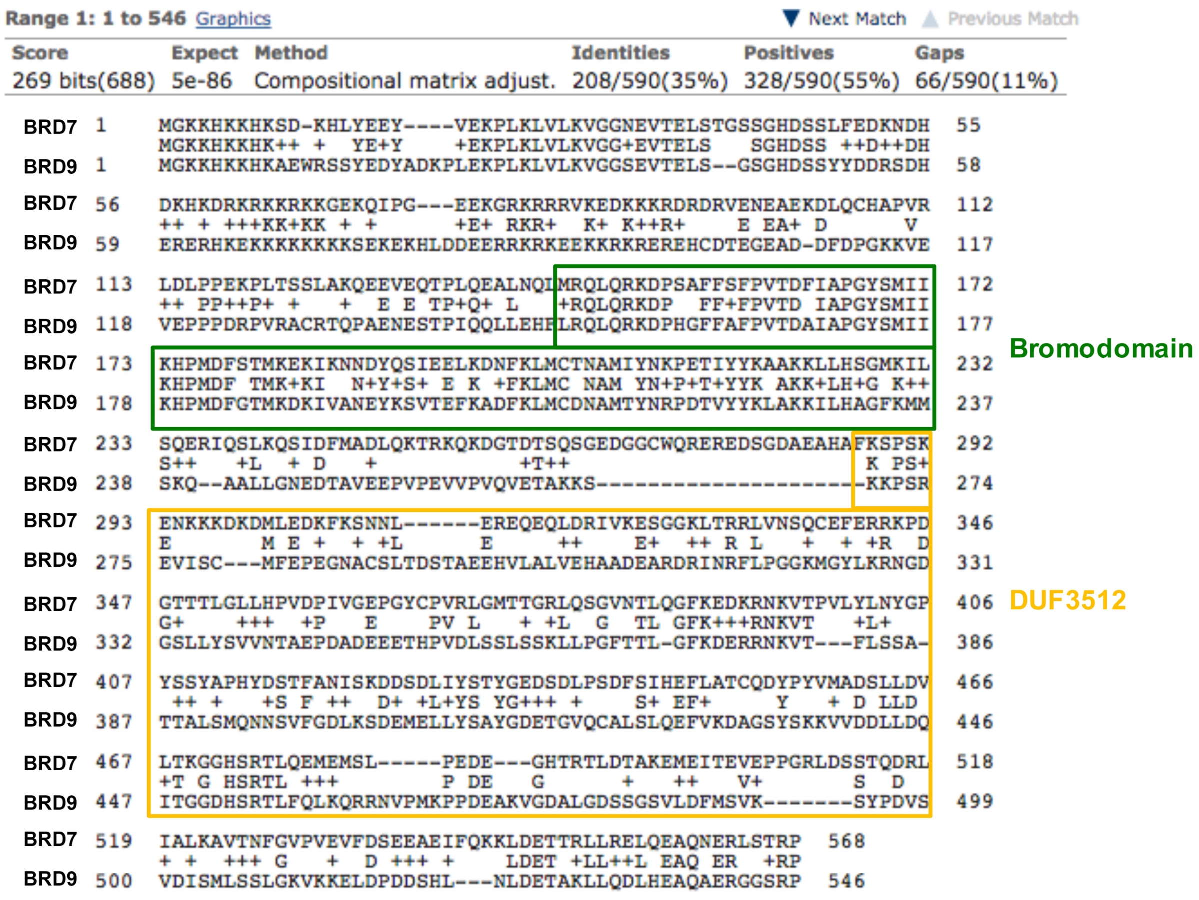

Supplementary Figure 3. Amino acid sequence comparison between BRD9 and BRD7. While the bromomodomains share high similarity, the DUF3512 domains are highly distinct.

Supplementary Figure 4

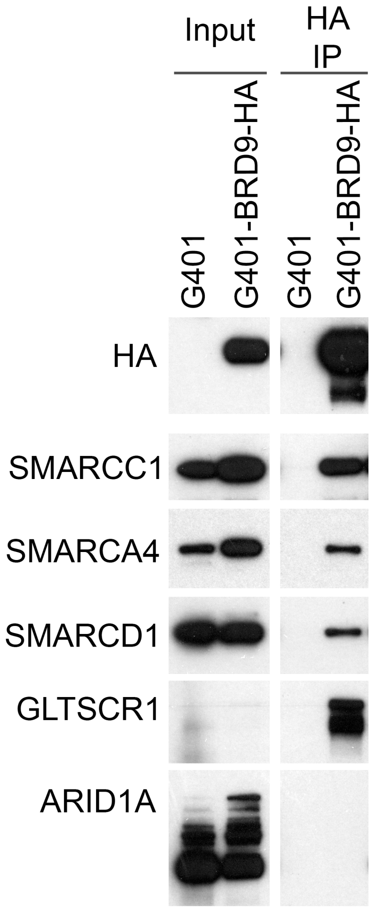

Supplementary Figure 4. C-terminal HA tagging does not affect the formation of BRD9-SWI/SNF subcomplex
HA-tag IP in parental G401 cells and G401-BRD9-HA (C-terminus) cells and Western blot of indicated subunits, showing the formation of BRD9-SWI/SNF subcomplex.

Supplementary Figure 5

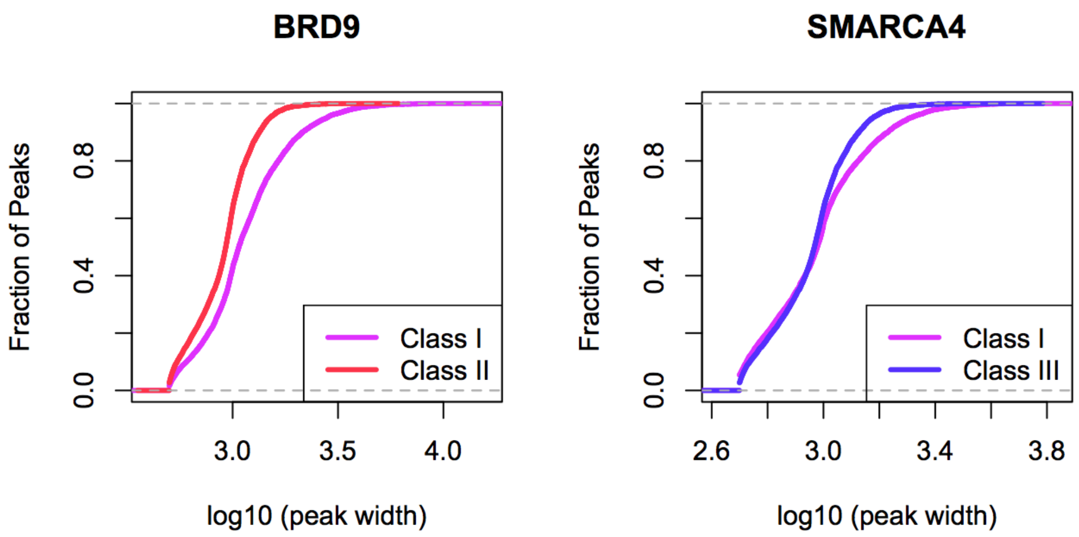

Supplementary Figure 5.

The cumulative distribution of BRD9 and SMARCA4 peak width in different peak categories in G401 cell.

Supplementary Figure 6

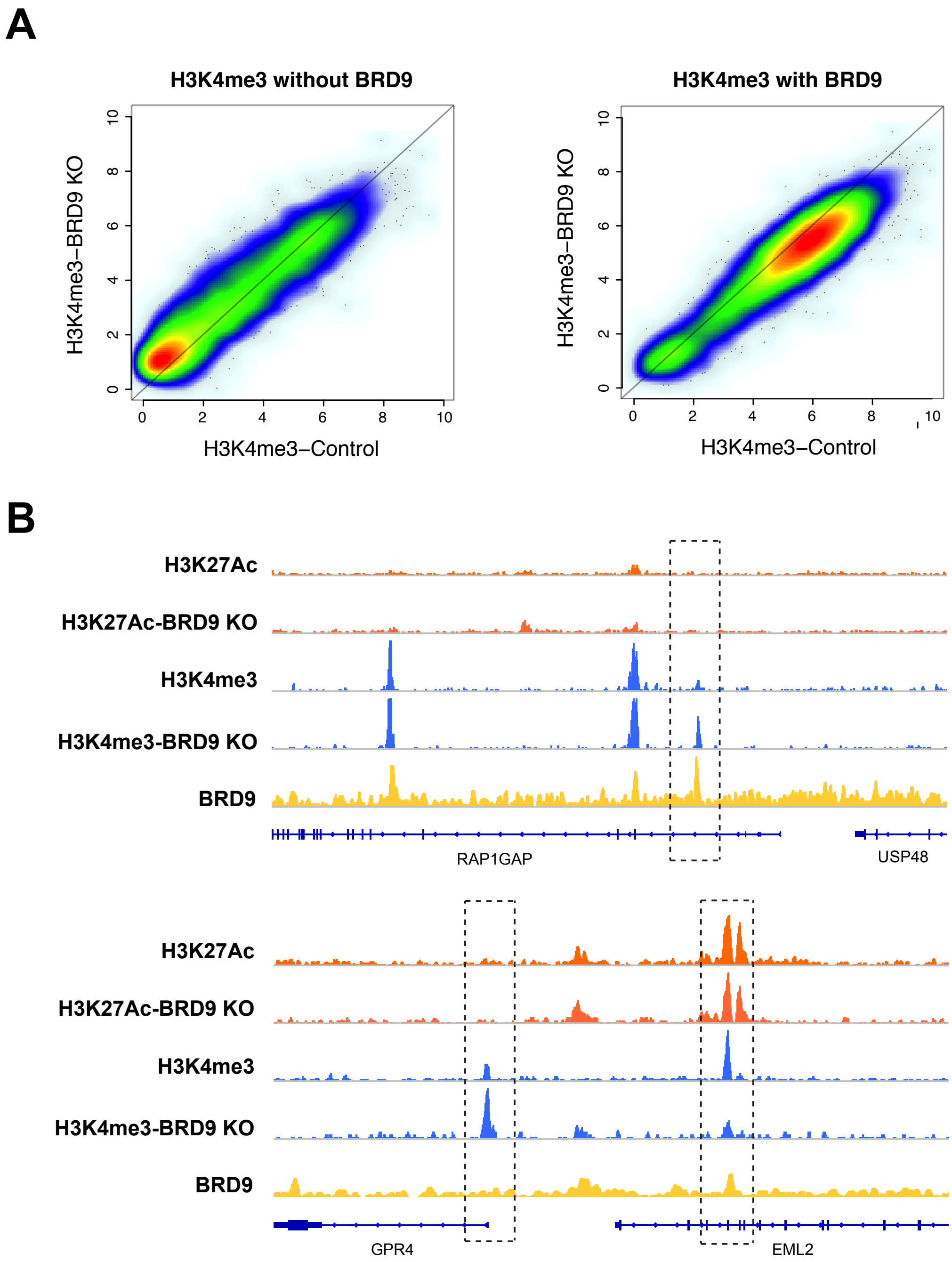

Supplementary Figure 6.
A. Comparison of the average H3K4me3 ChIP enrichment over H3K4me3 with BRD9 and without BRD9 binding sites between H3K4me3-Control and H3K4me3-BRD9 KO cells. Loss of BRD9 leads to bidirectional changes of H3K4me3. All experiments are performed in G401 cell.
B. Representative screenshots of H3K27ac, H3K4me3, and BRD9 in G401 cell without or with BRD9 deletion (KO) showing local changes of H3K4me3 upon BRD9 deletion: increased H3K4me3 signal (upper, lower left) and decreased K27Ac signal (lower right).

Supplementary Figure 7

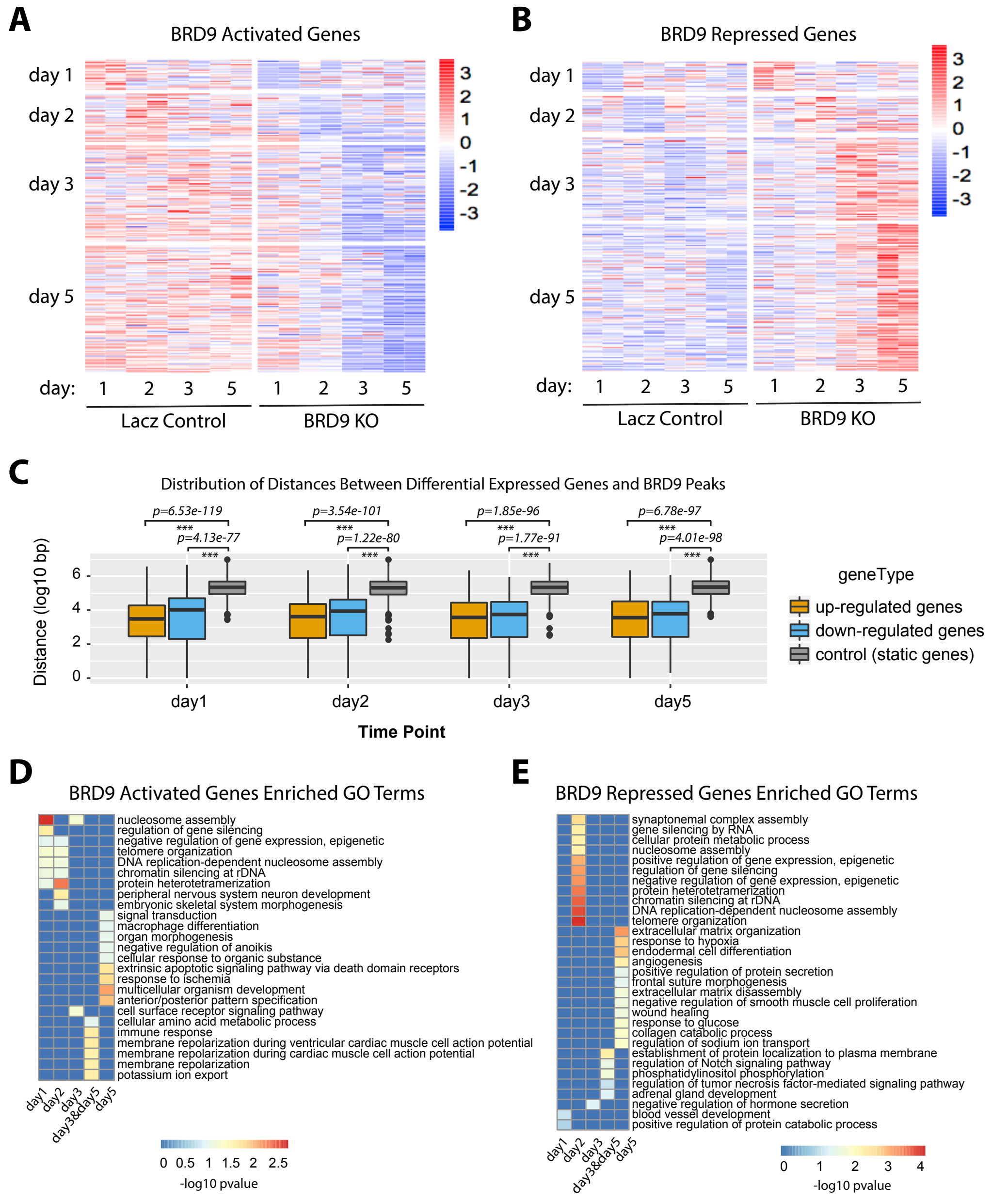

Supplementary Figure 7.
A-B. Scaled TPM of BRD9 (A) activated genes (expression decreased after KD BRD9) and **(B)** repressed genes (expression increased after KD BRD9) across different time points (day1, 2, 3, and 5) in Lacz control and BRD9 KO condition in G401 cell. Each time point has two biological replicates.
C. The distribution of distances between BRD9 up-regulated (orange), down-regulated (blue), static (used as control genes, grey) genes and BRD9 binding sites. P-values were calculated by Kolmogorov–Smirnov test.
D-E. The distribution of BRD9 **(D)** activated and **(E)** repressed genes enriched GO terms in different time points. Day3day5 presents genes differentially expressed in both day3 and day5.

Supplementary Figure 8

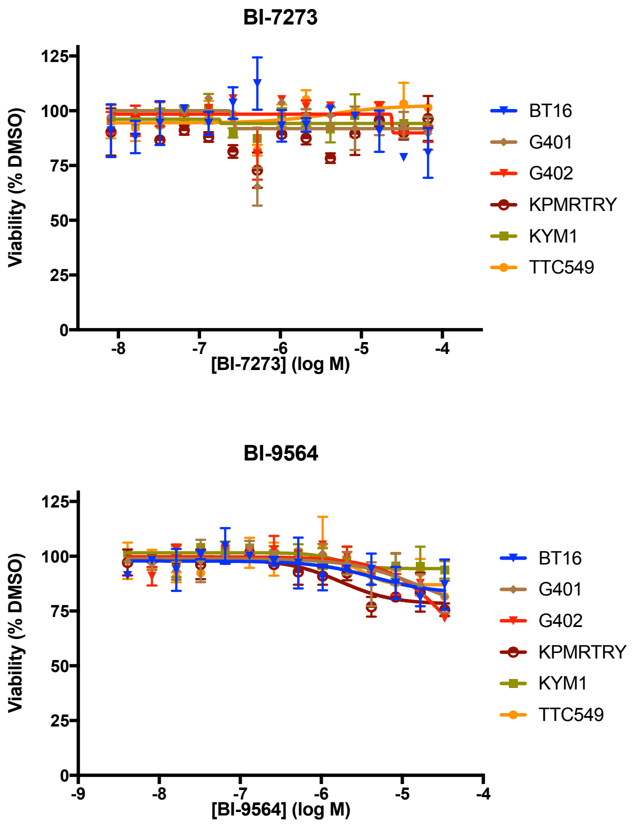

Supplementary Figure 8.
Treatment of BRD9 bromodomain specific inhibitors BI-7273, BI-9564 on a panel of RT cell lines showing that RTs are insensitive to BRD9 bromodomain inhibition.

Figure 2E

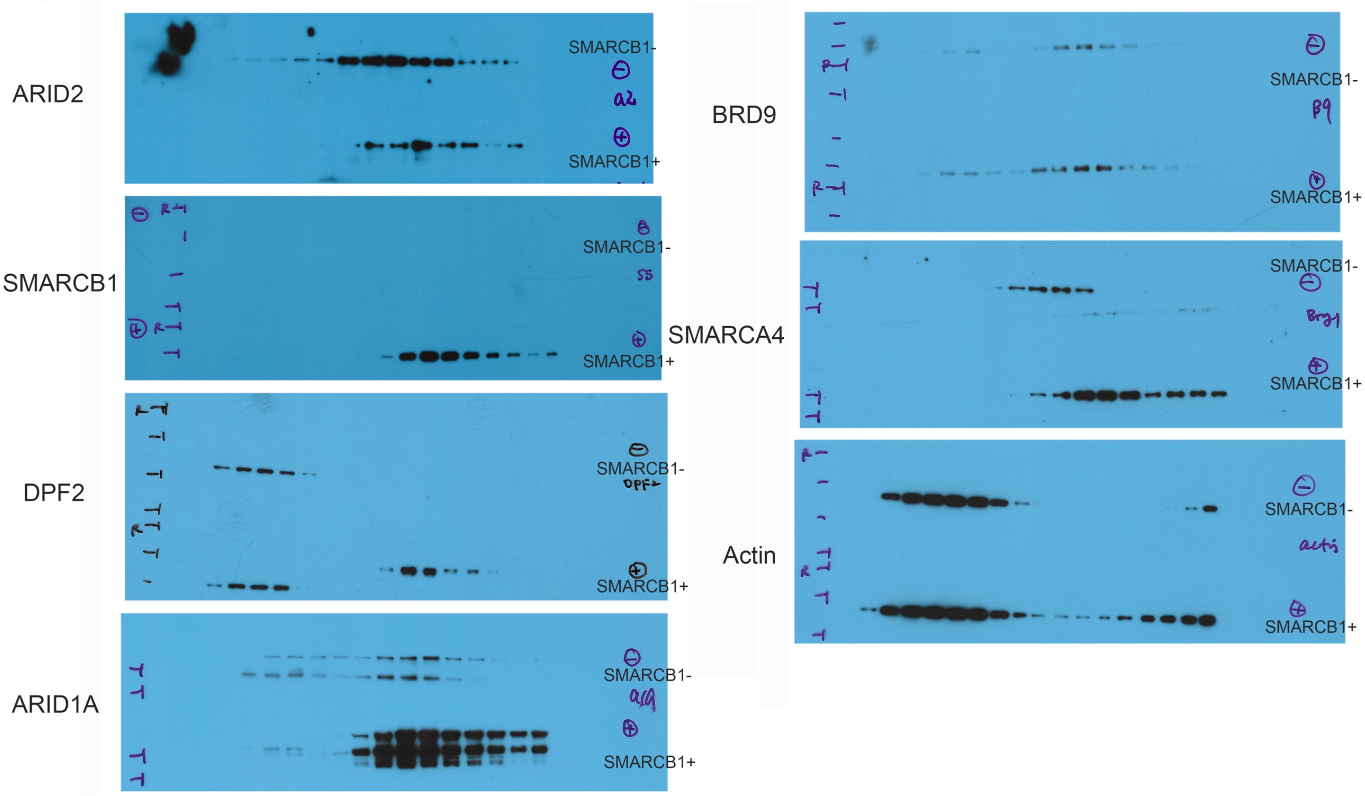

Raw Western blot scans related with Figure 2E

Figure 2F

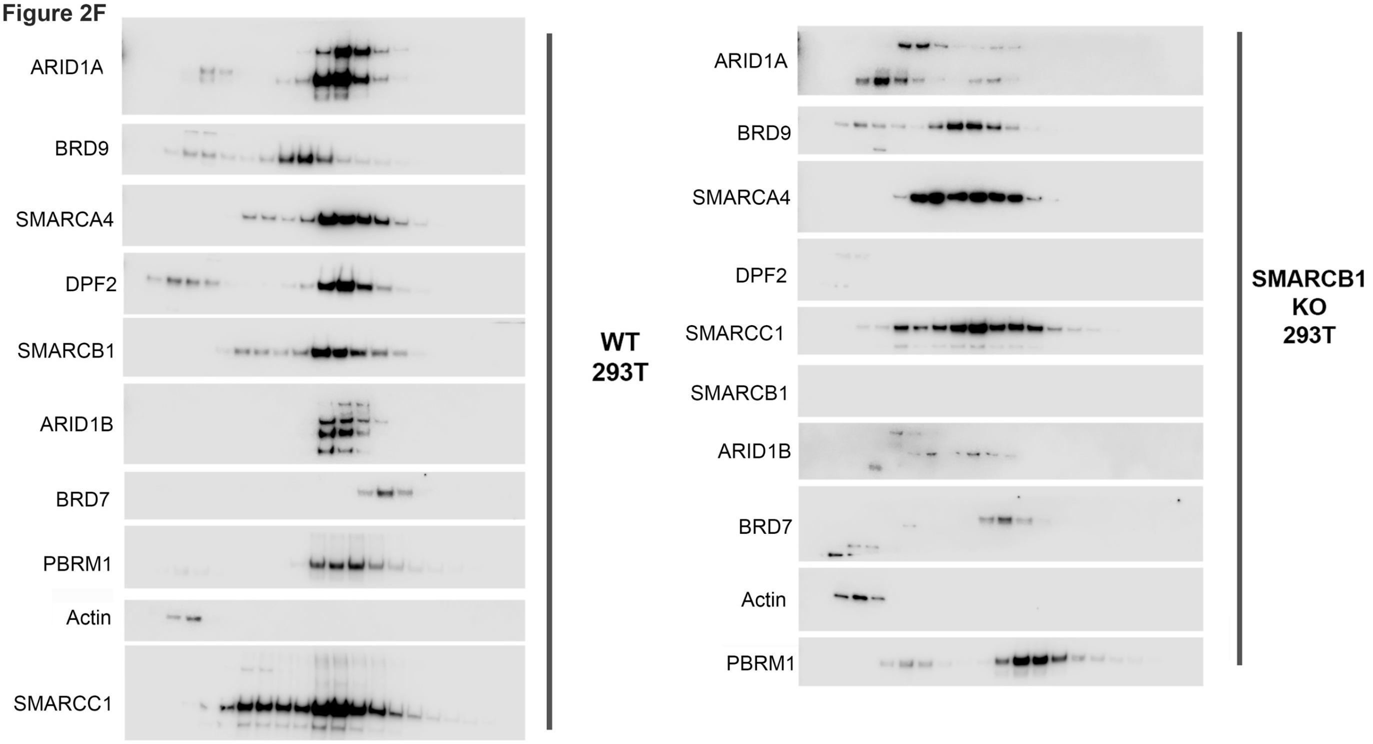

Supplementary Figure 2

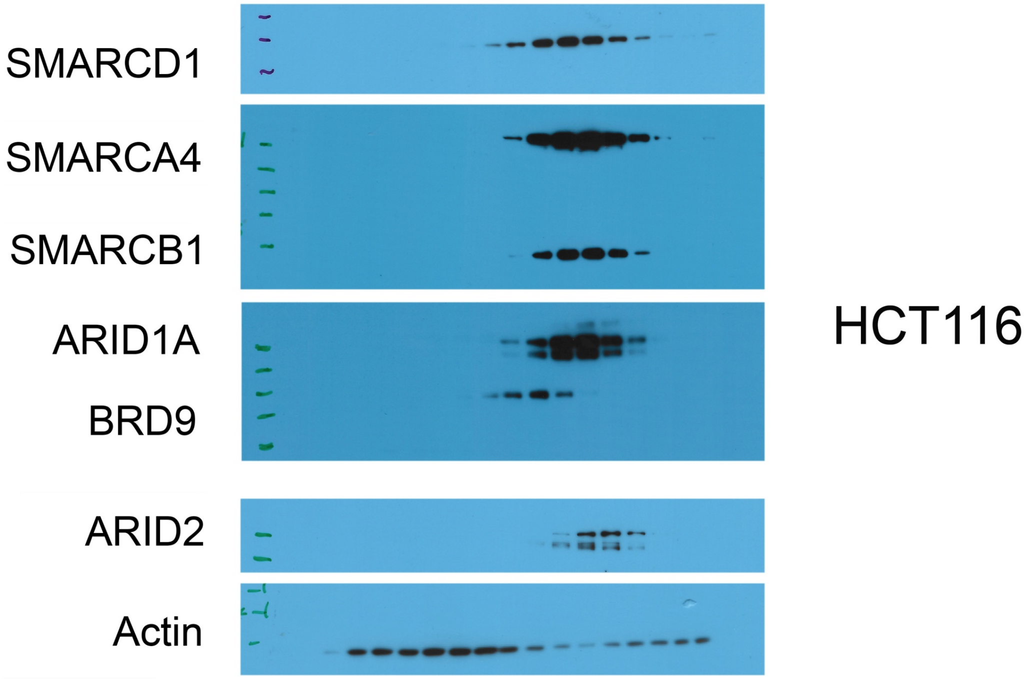

Raw Western blot scans related with Figure 2F and Supplementary Figure 2.

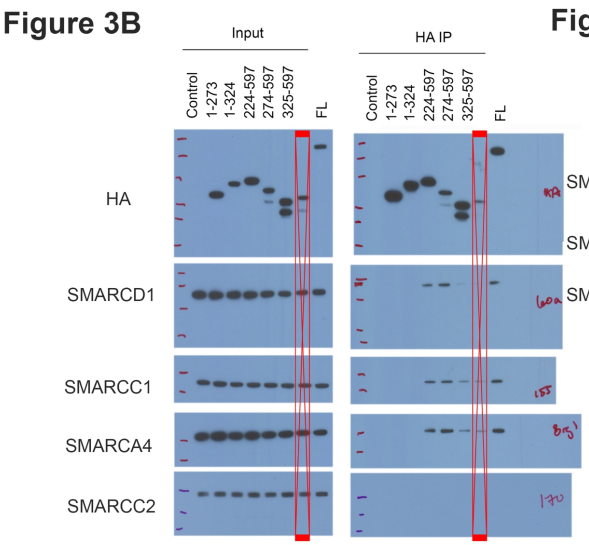

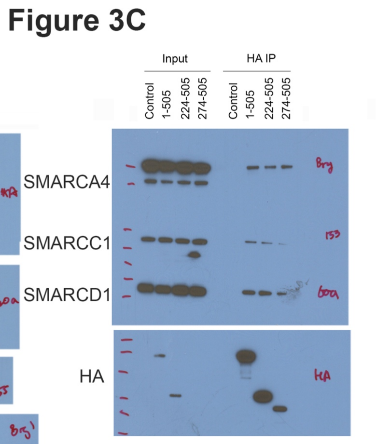

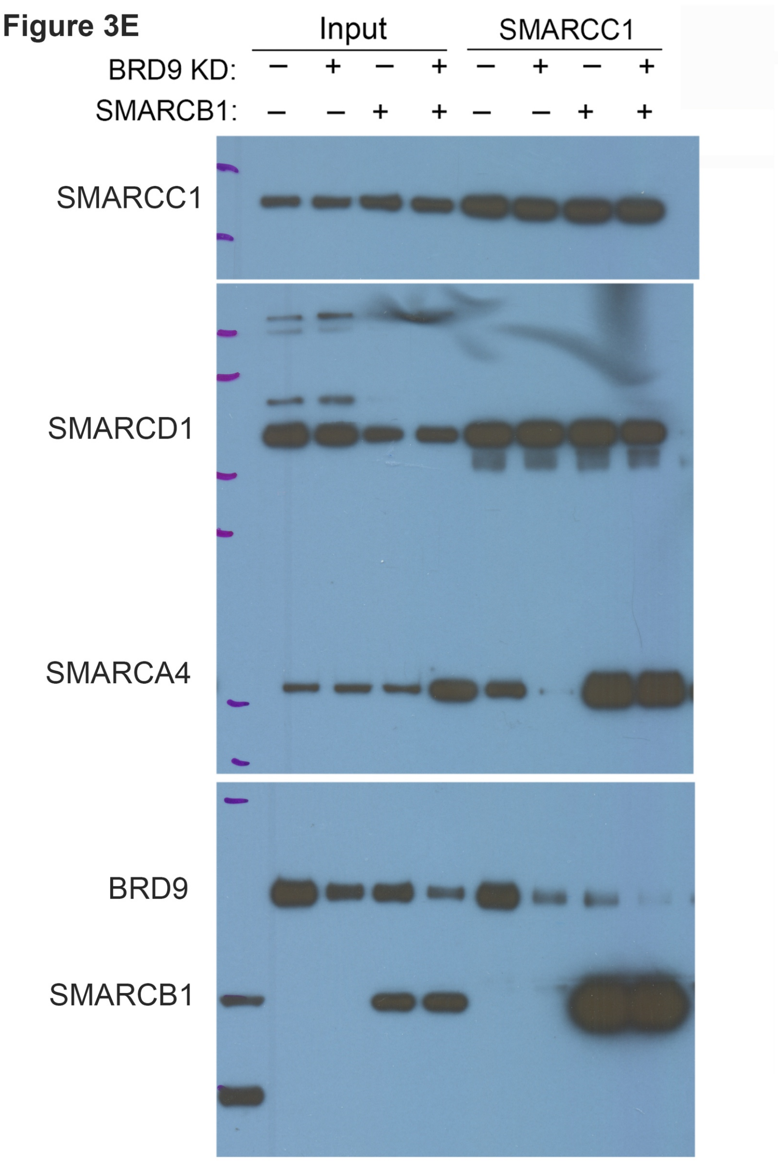

Raw Western blot scans related with Figure 3B,C and E

Figure 3D

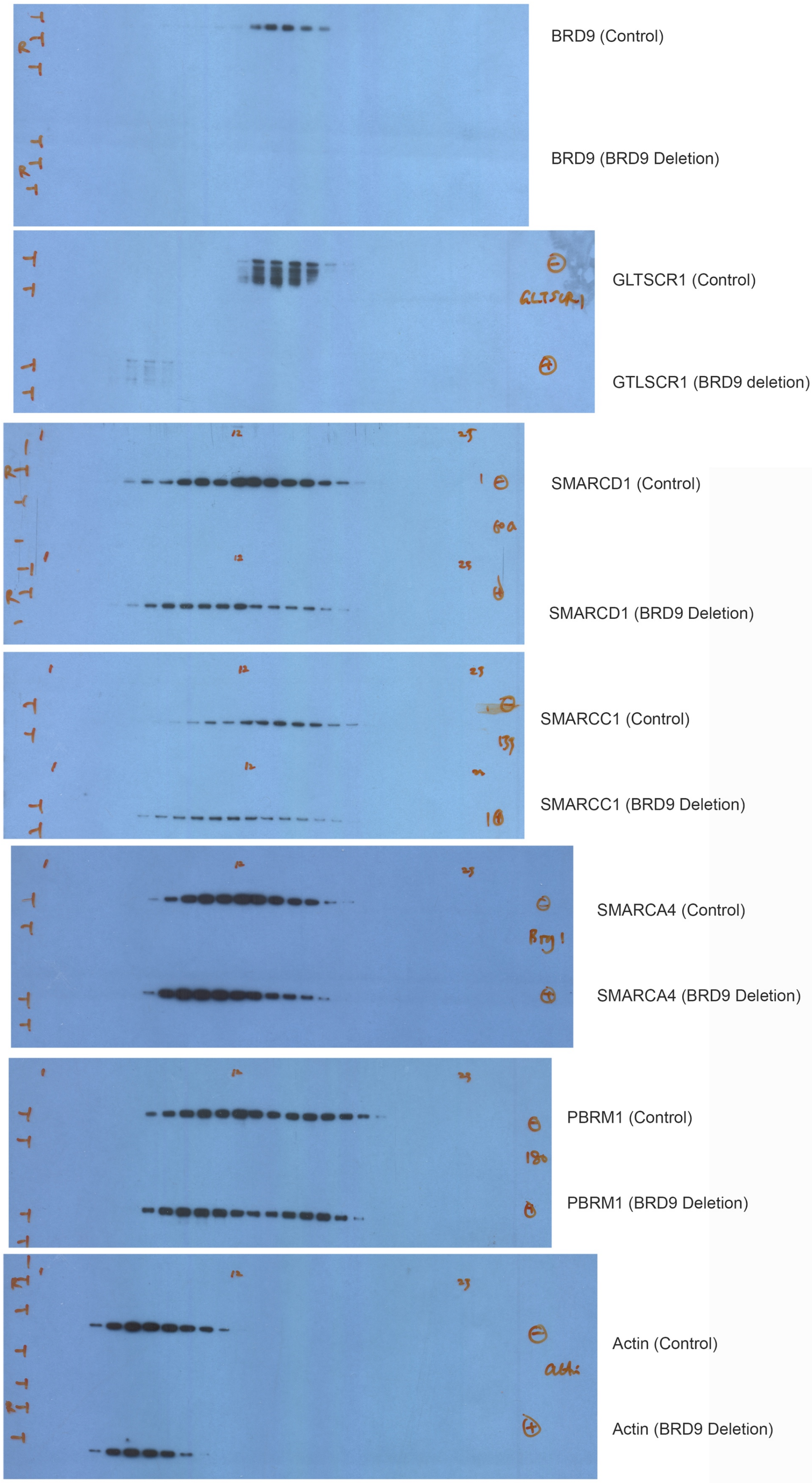

BRD9 (Control)

BRD9 (BRD9 Deletion)

GLTSCR1 (Control)

GTLSCR1 (BRD9 deletion)

SMARCD1 (Control)

SMARCD1 (BRD9 Deletion)

SMARCC1 (Control)

SMARCC1 (BRD9 Deletion)

SMARCA4 (Control)

SMARCA4 (BRD9 Deletion)

PBRM1 (Control)

PBRM1 (BRD9 Deletion)

Actin (Control)

Actin (BRD9 Deletion)

Raw Western blot scans related with Figure 3D
