# Supplementary material for: BRD9 defines a SWI/SNF sub-complex and constitutes a specific vulnerability in malignant rhabdoid tumors
Source: Nat Commun. 2019 Apr 23;10:1881. doi: 10.1038/s41467-019-09891-7 (PMC6479050; doi:10.1038/s41467-019-09891-7)
Supplement: Supplementary file 3 — Reporting Summary [file 41467_2019_9891_MOESM3_ESM.pdf]

nature research

Corresponding author(s): Charles W.M. Roberts

Reporting Summary

Nature Research wishes to improve the reproducibility of the work that we publish. This form provides structure for consistency and transparency in reporting. For further information on Nature Research policies, see Authors & Referees and the Editorial Policy Checklist.

Statistical parameters

When statistical analyses are reported, confirm that the following items are present in the relevant location (e.g. figure legend, table legend, main text, or Methods section).

n/a	Confirmed	
☐	☒	The exact sample size (*n*) for each experimental group/condition, given as a discrete number and unit of measurement
☐	☒	An indication of whether measurements were taken from distinct samples or whether the same sample was measured repeatedly
☐	☒	The statistical test(s) used AND whether they are one- or two-sided<br>*Only common tests should be described solely by name; describe more complex techniques in the Methods section.*
☒	☐	A description of all covariates tested
☒	☐	A description of any assumptions or corrections, such as tests of normality and adjustment for multiple comparisons
☐	☒	A full description of the statistics including central tendency (e.g. means) or other basic estimates (e.g. regression coefficient) AND variation (e.g. standard deviation) or associated estimates of uncertainty (e.g. confidence intervals)
☒	☐	For null hypothesis testing, the test statistic (e.g. *F*, *t*, *r*) with confidence intervals, effect sizes, degrees of freedom and *P* value noted<br>*Give P values as exact values whenever suitable.*
☒	☐	For Bayesian analysis, information on the choice of priors and Markov chain Monte Carlo settings
☒	☐	For hierarchical and complex designs, identification of the appropriate level for tests and full reporting of outcomes
☒	☐	Estimates of effect sizes (e.g. Cohen's *d*, Pearson's *r*), indicating how they were calculated
☐	☒	Clearly defined error bars<br>*State explicitly what error bars represent (e.g. SD, SE, CI)*

Our web collection on statistics for biologists may be useful.

Software and code

Policy information about availability of computer code

Data collection	SMARCA4, SMARCC1, H3K27Ac, and H3K4me3 ChIP-seq data from G401 cell were obtained from GSM1835876, GSM1835877, GSM1835878, GSM1835879, GSM1835880 respectively
Data analysis	ChIP-seq data sets were aligned to human genome hg19 using Bowtie, SPP package in R was used to identify the ChIP-seq enriched regions. Peaks genomic distribution was calculated via CEAS (http://liulab.dfci.harvard.edu/CEAS/) software. Motif finding was performed by MDSeqpos (https://bitbucket.org/cistrome/cistrome-applications-harvard/src/c477732c5c88/mdseqpos/) from Shirley Liu's lab. RNA-seq data sets were aligned to human genome hg19 using STAR. RSEM was used to do the transcript quantification, and differential expression analysis were performed with DESeq2.

For manuscripts utilizing custom algorithms or software that are central to the research but not yet described in published literature, software must be made available to editors/reviewers upon request. We strongly encourage code deposition in a community repository (e.g. GitHub). See the Nature Research guidelines for submitting code & software for further information.

April 2018

Data

Policy information about availability of data

All manuscripts must include a data availability statement. This statement should provide the following information, where applicable:

- Accession codes, unique identifiers, or web links for publicly available datasets
- A list of figures that have associated raw data
- A description of any restrictions on data availability

All raw and processed data are available in the GEO SuperSeries GSE120235. BRD9 defines a novel SWI/SNF sub-complex and constitutes a specific vulnerability in malignant rhabdoid tumors. Reviewers can access these data via secure token: wrqpyigqdxofxsb

Field-specific reporting

Please select the best fit for your research. If you are not sure, read the appropriate sections before making your selection.

☒ Life sciences ☐ Behavioural & social sciences ☐ Ecological, evolutionary & environmental sciences

For a reference copy of the document with all sections, see nature.com/authors/policies/ReportingSummary-flat.pdf

Life sciences study design

All studies must disclose on these points even when the disclosure is negative.

Sample size	Experiments were performed as two replicates to ensure biological reproducibility. No power analysis was initially performed to determine the required sample size
Data exclusions	No data were excluded from analysis
Replication	experimental findings were reliably reproduced
Randomization	No randomization was performed. Our study involved the characterization of known cancer cell lines, so randomization is not possible
Blinding	The investigators were not blinded to analysis of data from cell lines. No animal or human subjects were used.

Reporting for specific materials, systems and methods

Materials & experimental systems

n/a	Involved in the study
☐	☐ Unique biological materials
☐	☒ Antibodies
☐	☒ Eukaryotic cell lines
☐	☐ Palaeontology
☐	☐ Animals and other organisms
☐	☐ Human research participants

Methods

n/a	Involved in the study
☐	☒ ChIP-seq
☐	☐ Flow cytometry
☐	☐ MRI-based neuroimaging

Unique biological materials

Policy information about availability of materials

Obtaining unique materials	*Describe any restrictions on the availability of unique materials OR confirm that all unique materials used are readily available from the authors or from standard commercial sources (and specify these sources).*

Antibodies

Antibodies used	BRD9 (Bethyl Laboratories: A303-781A); SMARCC1/BAF155 (Santa Cruz: sc9746); ARID1A (Santa Cruz: sc-32761 for immunoprecipitation; Cell Signaling Technology: 12354 for immunoblotting); SMARCA4/BRG1 (Santa Cruz: sc17796); GLTSCR1 (Santa Cruz: sc-515086); SMARCC2/BAF170 (Bethyl Laboratories: A301-039A); SMARCD1/BAF60A (Bethyl Laboratories: A301-595A); SMARCE1/ BAF57 (Bethyl Laboratories: A300-810A); SMARCB1/SNF5 (Bethyl Laboratories: A301-087A); ACTL6A/

	BAF53A (Bethyl Laboratories: A301-391A); PBRM1 (Bethyl Laboratories: A301-591A); HA-tag (Cell Signaling Technology, 3724); ACTIN (Cell Signaling Technology: 5125)
Validation	All commercially obtained antibodies were used according to manufacturer's instructions.

Eukaryotic cell lines

Policy information about cell lines

Cell line source(s)	G401, H1299, HCT116 and ES-2 cell lines were purchased from American Type Culture Collection (ATCC). BT16, TOV21G, TTC709, TTC642 and TTC549 cells were maintained in the lab. KP-MRT-RY was a kind gift from Yasumichi Kuwahara at the Kyoto Prefectural University of Medicine.
Authentication	The identities of the cell lines were validated by SNP fingerprinting as described previously (Rees et al., Nat. Chem. Biol. (2016). doi:10.1038/nchembio.1986).
Mycoplasma contamination	Cell lines were tested for Mycoplasma negative (Rees et al., Nat. Chem. Biol. (2016). doi:10.1038/nchembio.1986).
Commonly misidentified lines (See ICLAC register)	*Name any commonly misidentified cell lines used in the study and provide a rationale for their use.*

Palaeontology

Specimen provenance	*Provide provenance information for specimens and describe permits that were obtained for the work (including the name of the issuing authority, the date of issue, and any identifying information).*
Specimen deposition	*Indicate where the specimens have been deposited to permit free access by other researchers.*
Dating methods	*If new dates are provided, describe how they were obtained (e.g. collection, storage, sample pretreatment and measurement), where they were obtained (i.e. lab name), the calibration program and the protocol for quality assurance OR state that no new dates are provided.*

☐ Tick this box to confirm that the raw and calibrated dates are available in the paper or in Supplementary Information.

Animals and other organisms

Policy information about studies involving animals; ARRIVE guidelines recommended for reporting animal research

Laboratory animals	*For laboratory animals, report species, strain, sex and age OR state that the study did not involve laboratory animals.*
Wild animals	*Provide details on animals observed in or captured in the field; report species, sex and age where possible. Describe how animals were caught and transported and what happened to captive animals after the study (if killed, explain why and describe method; if released, say where and when) OR state that the study did not involve wild animals.*
Field-collected samples	*For laboratory work with field-collected samples, describe all relevant parameters such as housing, maintenance, temperature, photoperiod and end-of-experiment protocol OR state that the study did not involve samples collected from the field.*

Human research participants

Policy information about studies involving human research participants

Population characteristics	*Describe the covariate-relevant population characteristics of the human research participants (e.g. age, gender, genotypic information, past and current diagnosis and treatment categories). If you filled out the behavioural & social sciences study design questions and have nothing to add here, write "See above."*
Recruitment	*Describe how participants were recruited. Outline any potential self-selection bias or other biases that may be present and how these are likely to impact results.*

ChIP-seq

Data deposition

☒ Confirm that both raw and final processed data have been deposited in a public database such as GEO.

☒ Confirm that you have deposited or provided access to graph files (e.g. BED files) for the called peaks.

Data access links<br>*May remain private before publication.*	https://www.ncbi.nlm.nih.gov/geo/query/acc.cgi?acc=GSE120235
Files in database submission	GSM3396517 G401_BRD9KO_rep1

Files in database submission	GSM3396518 G401_BRD9KO_rep2<br>GSM3396519 G401_LacZ_rep1<br>GSM3396520 G401_LacZ_rep2<br>GSM3396521 G401_HAIP_BRD9_rep1<br>GSM3396522 G401_HAIP_BRD9_rep2<br>GSM3396523 G401_HAIP_Input_rep1<br>GSM3396524 G401_BRD9KO_H3K27Ac_rep1<br>GSM3396525 G401_BRD9KO_H3K27Ac_rep2<br>GSM3396526 G401_BRD9KO_H3K4me3_rep1<br>GSM3396527 G401_BRD9KO_Input_rep1<br>GSM3396528 G401_control_H3K27Ac_rep1<br>GSM3396529 G401_control_H3K27Ac_rep2<br>GSM3396530 G401_control_H3K4me3_rep1<br>GSM3396531 G401_control_Input_rep1
Genome browser session (e.g. UCSC)	*Provide a link to an anonymized genome browser session for "Initial submission" and "Revised version" documents only, to enable peer review. Write "no longer applicable" for "Final submission" documents.*

Methodology

Replicates	BRD9: 2 reps; H3K27Ac BRD9KO: 2 reps; H2K27Ac Control: 2 reps; H3K4me3 BRD9KO: 1 rep; H3K4me3 Control: 1 rep; Input: 2 reps
Sequencing depth	Total Read: BRD9_rep1: 5,091,779, BRD9_rep1: 40,486,409, H3K27Ac_BRD9KO_rep1: 22,173,570, H3K27Ac_BRD9KO_rep2: 18,399,544, H3K4me3_BRD9KO_rep1: 21,410,342, H3K27Ac_Control_rep1: 23,222,077, H3K27Ac_Control_rep2: 29,481,289, H3K4me3_Control_rep1: 20,907,367<br>Mapped Reads: BRD9_rep1: 4,337,395, BRD9_rep1: 34,503,090, H3K27Ac_BRD9KO_rep1: 19,301,317, H3K27Ac_BRD9KO_rep2: 15,800,306, H3K4me3_BRD9KO_rep1: 18,449,648, H3K27Ac_Control_rep1: 19,956,251, H3K27Ac_Control_rep2: 25,321,909, H3K4me3_Control_rep1: 18,162,954<br>Read length: 75bp singled-end sequencing
Antibodies	HA-tag (Cell Signaling Technology: 3724); H3K27Ac (Cell Signaling Technology: 8173) H3K4me3 (Cell Signaling Technology: 9751)
Peak calling parameters	Mapping parameter: –m 1 --best in Bowtie<br>Peak calling parameters: window.size=500, z.thr=4 in SPP
Data quality	FastQC was performed to do the sequencing data quality control.<br>BRD9 Peaks: 13,476 (cutoff of z.thr=5 used here, z.thr is the Z-score corresponding to the Poisson ratio threshold used to flag significantly) enriched windows.<br>H3K27Ac_BRD9KO_peaks: 24,713 (z.thr=4), H3K27Ac_Control peaks: 20,639 (z.thr=4)<br>H3K4me3_BRD9KO peaks: 15,435 (z.thr=4), H3K4me3_Control peaks: 14,281 (z.thr=4)
Software	Peaks genomic distribution was calculated via CEAS (http://liulab.dfci.harvard.edu/CEAS/) software. Motif finding was performed by MDSeqpos (https://bitbucket.org/cistrome/cistrome-applications-harvard/src/c477732c5c88/mdseqpos/) from Shirley Liu's lab.

Flow Cytometry

Plots

Confirm that:

- [ ] The axis labels state the marker and fluorochrome used (e.g. CD4-FITC).
- [ ] The axis scales are clearly visible. Include numbers along axes only for bottom left plot of group (a 'group' is an analysis of identical markers).
- [ ] All plots are contour plots with outliers or pseudocolor plots.
- [ ] A numerical value for number of cells or percentage (with statistics) is provided.

Methodology

Sample preparation	*Describe the sample preparation, detailing the biological source of the cells and any tissue processing steps used.*
Instrument	*Identify the instrument used for data collection, specifying make and model number.*
Software	*Describe the software used to collect and analyze the flow cytometry data. For custom code that has been deposited into a community repository, provide accession details.*
Cell population abundance	*Describe the abundance of the relevant cell populations within post-sort fractions, providing details on the purity of the samples and how it was determined.*

Gating strategy — *Describe the gating strategy used for all relevant experiments, specifying the preliminary FSC/SSC gates of the starting cell population, indicating where boundaries between "positive" and "negative" staining cell populations are defined.*

☐ Tick this box to confirm that a figure exemplifying the gating strategy is provided in the Supplementary Information.

Magnetic resonance imaging

Experimental design

Design type — *Indicate task or resting state; event-related or block design.*

Design specifications — *Specify the number of blocks, trials or experimental units per session and/or subject, and specify the length of each trial or block (if trials are blocked) and interval between trials.*

Behavioral performance measures — *State number and/or type of variables recorded (e.g. correct button press, response time) and what statistics were used to establish that the subjects were performing the task as expected (e.g. mean, range, and/or standard deviation across subjects).*

Acquisition

Imaging type(s) — *Specify: functional, structural, diffusion, perfusion.*

Field strength — *Specify in Tesla*

Sequence & imaging parameters — *Specify the pulse sequence type (gradient echo, spin echo, etc.), imaging type (EPI, spiral, etc.), field of view, matrix size, slice thickness, orientation and TE/TR/flip angle.*

Area of acquisition — *State whether a whole brain scan was used OR define the area of acquisition, describing how the region was determined.*

Diffusion MRI ☐ Used ☐ Not used

Preprocessing

Preprocessing software — *Provide detail on software version and revision number and on specific parameters (model/functions, brain extraction, segmentation, smoothing kernel size, etc.).*

Normalization — *If data were normalized/standardized, describe the approach(es): specify linear or non-linear and define image types used for transformation OR indicate that data were not normalized and explain rationale for lack of normalization.*

Normalization template — *Describe the template used for normalization/transformation, specifying subject space or group standardized space (e.g. original Talairach, MNI305, ICBM152) OR indicate that the data were not normalized.*

Noise and artifact removal — *Describe your procedure(s) for artifact and structured noise removal, specifying motion parameters, tissue signals and physiological signals (heart rate, respiration).*

Volume censoring — *Define your software and/or method and criteria for volume censoring, and state the extent of such censoring.*

Statistical modeling & inference

Model type and settings — *Specify type (mass univariate, multivariate, RSA, predictive, etc.) and describe essential details of the model at the first and second levels (e.g. fixed, random or mixed effects; drift or auto-correlation).*

Effect(s) tested — *Define precise effect in terms of the task or stimulus conditions instead of psychological concepts and indicate whether ANOVA or factorial designs were used.*

Specify type of analysis: ☐ Whole brain ☐ ROI-based ☐ Both

Statistic type for inference (See Eklund et al. 2016) — *Specify voxel-wise or cluster-wise and report all relevant parameters for cluster-wise methods.*

Correction — *Describe the type of correction and how it is obtained for multiple comparisons (e.g. FWE, FDR, permutation or Monte Carlo).*

Models & analysis

n/a	Involved in the study
☐	☐ Functional and/or effective connectivity
☐	☐ Graph analysis
☐	☐ Multivariate modeling or predictive analysis

Functional and/or effective connectivity	*Report the measures of dependence used and the model details (e.g. Pearson correlation, partial correlation, mutual information).*
Graph analysis	*Report the dependent variable and connectivity measure, specifying weighted graph or binarized graph, subject- or group-level, and the global and/or node summaries used (e.g. clustering coefficient, efficiency, etc.).*
Multivariate modeling and predictive analysis	*Specify independent variables, features extraction and dimension reduction, model, training and evaluation metrics.*
