# Supplementary material for: BRD9 defines a SWI/SNF sub-complex and constitutes a specific vulnerability in malignant rhabdoid tumors
Source: Nat Commun. 2019 Apr 23;10:1881. doi: 10.1038/s41467-019-09891-7 (PMC6479050; doi:10.1038/s41467-019-09891-7)
Supplement: Supplementary file 2 — Peer Review File [file 41467_2019_9891_MOESM2_ESM.pdf]

Reviewers' comments:

Reviewer #1 (Remarks to the Author):

This is an excellent study from the Roberts laboratory, describing a critical role of BRD9 in malignant rhabdoid tumor. The authors use a genomewide CRISPR screen to identify a critical role for BRD9 in MRT cell lines, which is subsequently validated. The go on to characterize a novel BAF complex assembly that includes BRD9, GLTSCR1, SMARCA2/4, SMARCC1, whereas other BAF subunits (e.g. ARID1A and SMARCB1) are lacking. Moreover, they go on the map the critical domain of BRD9 involved in the formation of this complex and they show that loss of BRD9 disrupts this complex. Finally, an epigenomics analysis revealed the critical cis elements controlled by this factor.

Overall, this is a comprehensive body of work that validates a novel target in MRT as well a novel variant of the BAF complex. The findings presented in this study are convincing, and I believe this study is a strong candidate for publication in Nature Communications. I have only a couple suggestions below that I believe would improve the current study.

1) The CRISPR screen results of BRD9 dependency were only validated in two of the MRT cell lines. The authors should validate this finding in at least two addition MRT lines, and using at least 2 additional BRD9 independent non-MRT cell lines as a control for the specificity.

Reviewer #2 (Remarks to the Author):

This manuscript describes the characterisation of BRD9, a protein found in a CRISPR screen to be required for survival of SMARCB1-mutant rhabdoid tumour cell lines. The study provides a good biochemical description of the BRD9-containing complex, which also contains some BAF complex components, and how this differs from other BAF-related complexes. The molecular biology describing what BRD9 actually does, or what the specific function of this complex might be, are much weaker with no definitive experiments performed to tell us what the complex does in healthy cells or why its absence causes SMARCB1-mutant tumour cells to die.

There is a general lack of explanation of what is being plotted in figures, size marker info on westerns, and information about replicate numbers. Quite often I have no idea what is being plotted in graphs. I also find it difficult to follow which cells are being used for which analyses. Were the RNA-seq and ChIP-seq experiments all performed in G401 cells with exogenous SMARCB1 expression? How many replicates were performed for each high-throughput sequencing experiment?

ChIP-seq: Class I sites are presumably those bound by this novel BRD9/SMARCA4 complex. What do Class II sites represent? The biochemical evidence so far indicates that BRD9 forms a novel complex with some BAF components as depicted in Figure 2D. These Class II sites, however, appear to be bound by BRD9 without SMARCA4 or SMARCC1. What do we make of these? Are the authors arguing that BRD9 also functions outside of this novel complex? The glycerol gradient gels do not support an idea that BRD9 can also function on its own.

The final section aims to identify “direct targets” of BRD9 using a published algorithm to try to work out what kinds of genes may be regulated by BRD9. They identify some genes and perform GO analyses, but in the end the conclusion is that BRD9 might somehow be involved in activating genes associated with apoptosis and in “additional biological functions”. We really haven’t learned much from this analysis. What are the authors trying to demonstrate here? If the aim is to learn why BRD9/SMARCB1 double null cells die, then it would be interesting to compare gene expression in both single mutants with a double mutant (e.g. a short time after loss of one of the proteins). If the

question is rather "which genes show a transcriptional response to the loss of BRD9?" then it would be necessary to carry out an inducible deletion or depletion to distinguish primary transcriptional changes from secondary, tertiary, or adaptive transcriptional changes.

Minor comments:

Figure 1C: I think this is mislabelled: it looks like each panel has shRNA 1 and 2.

Figure 1F: why are there residual protein bands in the G401 lanes if this is a knockout? Are these clonal lines?

Lines 154-157 and Figure 2E: PBRM1 is widely distributed across the gradient gel, so it is incorrect to state that BRD9 and GLTSCR1 appear before PBRM1

Line 165: The authors show that BRD9 is associating with other proteins individually in this assay, not that it forms a subcomplex (i.e. that it associates with all of them at the same time).

Line 173 and Figure 3D: There is almost no signal for GLTSCR1 on this blot. Is it expressed in these cells? It would be useful to have an input lane to demonstrate this. There is almost no signal in the input lanes of Supplementary Fig 3, again is this just not very well expressed in these cells?

Figure 5A: Would be useful to include something that does show a change just for comparison, such as (presumably) unreconstituted G401 cells. Many of these lanes are overexposed so it would be difficult to detect modest changes.

Lines 226-227: I don't agree that the identification of DNA binding motifs for ETS family proteins at Class II sites, which are not bound by other SWI/SNF family members and occur overwhelmingly at promoters is "…consistent with a recent report that the SWI/SNF complex interacts with EWS-FLI1 and targets tumor-specific enhancers…."

Reviewer #3 (Remarks to the Author):

Alpsoy and Dykhuizen (2018, JBC) previously demonstrated the existence of a sub-complex of BAF complexes which they termed "GBAF", for its unique incorporation of GLTSCR1/1L and BRD9, and the absence of core BAF components ARID1A/B, BAF57 and BAF47 (SMARCB1). In this manuscript, Wang et al., coming from an unbiased approach arising from the Achilles Project, report that SMARCB1-deficient rhabdoid tumors (RT) are differentially sensitive to the loss of BRD9 (Fig 1), suggesting that the GBAF complex might be involved in the RT malignancy.

The authors reproduce Dykhuizen's findings and confirm the existence of a BAF subcomplex defined by BRD9 and GLTSCR1. While this complex is present in both WT and SMARCB1-deficient RT cell lines, the authors propose but do not show conclusive proof that the GBAF complex dominates in SMARCB1-deficient RT cell lines. They further propose that this complex becomes essential in the absence or reduction of canonical SMARCB1-containing complexes, explaining the exquisite sensitivity of SMARCB1-deficient RT lines to BRD9-inhibition. Reduction of BRD9 causes the dissociation of complexes and subcomplexes, and re-expression of SMARCB1 induces re-assembly of canonical complexes.

In RT cell lines, the authors demonstrate that GBAF complexes have similar and distinct gene regulatory functions compared to canonical BAF complexes, and the transcriptional regulatory profile of BRD9 is consistent with a program that drives cell cycle and survival.

The findings presented in these paper are of interest due to the potential clinical utility of inhibiting BRD9 in RT based on its crucial role in BAF complexes. However, the authors have not demonstrated convincing evidence that BRD9 complex predominates in the absence of SMARCB1, which should be provided before the paper is suitable for publication.

Major comments:
1) The conclusion that SMARCB1 loss drives the formation of alternative BRD9 complexes hinges on Figure 3E-F which are artificial manipulations of SMARCB1 expression. In my opinion, conclusive evidence that BRD9 complexes predominate in the absence of SMARCB1 include : a) glycerol gradients in WT and SMARCB1-mutant cell lines to see size redistribution of core subunits which in the SMARCB1-mutant lines presumably assemble with BRD9 complexes that are smaller. b) Immuno-depletion assays to show that in SMARCB1 mutant lines, BRD9 IP can immune-deplete core subunits which it assembles with to a greater extent than in WT lines.

2) Since the most interesting conclusion of the paper – that BRD9 inhibition might have therapeutic effect in RT and that this is not an in vitro phenomenon, the authors should attempt some xenograft experiments in the presence and absence of BRD9 knockdown. If the BRD9 degradation molecules are available, it would be even better to see if these have any efficacy as proposed in the concluding statement.

Minor comment :
1) Please refer to the reported subcomplex as GBAF since it appears to be the same sub-complex reported by Alpsoy.

Response to reviewers' comments:

We thank the reviewers for their careful reading of the manuscript and their many suggestions. We have revised the manuscript substantially in response to the comments, and we believe it has become a much stronger manuscript.

Reviewer #1:

This is an excellent study from the Roberts laboratory, describing a critical role of BRD9 in malignant rhabdoid tumor. The authors use a genomewide CRISPR screen to identify a critical role for BRD9 in RT cell lines, which is subsequently validated. The go on to characterize a novel BAF complex assembly that includes BRD9, GLTSCR1, SMARCA2/4, SMARCC1, whereas other BAF subunits (e.g. ARID1A and SMARCB1) are lacking. Moreover, they go on the map the critical domain of BRD9 involved in the formation of this complex and they show that loss of BRD9 disrupts this complex. Finally, an epigenomics analysis revealed the critical cis elements controlled by this factor.

Overall, this is a comprehensive body of work that validates a novel target in RT as well a novel variant of the BAF complex. The findings presented in this study are convincing, and I believe this study is a strong candidate for publication in Nature Communications. I have only a couple suggestions below that I believe would improve the current study.

The CRISPR screen results of BRD9 dependency were only validated in two of the RT cell lines. The authors should validate this finding in at least two addition RT lines, and using at least 2 additional BRD9 independent non-RT cell lines as a control for the specificity.

Following Reviewer 1's advice, we validated the results of our BRD9 dependency screen in additional RT and non-RT cell lines (**Supplementary Figure 1A-B**). Now incorporated into **Supplementary Figure 1A-B**, we show that deletion of BRD9 has no effect on pediatric Ewing Sarcoma cell line EW8 or upon pancreatic cell line PANC-1 (both are SMARCB1 wild type); however, loss of BRD9 impairs the proliferation of TTC642 (SMARCB1-mutant RT cell line), and the degree of proliferation inhibition correlates with the BRD9 CRISPR efficiency. We observed modest proliferation inhibition in another RT cell line—KD, due to low BRD9 CRIPSR editing efficiency. Taken together, these further support our conclusion that that BRD9 is a specific dependency for SMARCB1-deficient cancer cells.

Reviewer #2 (Remarks to the Author):

This manuscript describes the characterisation of BRD9, a protein found in a CRISPR screen to be required for survival of SMARCB1-mutant rhabdoid tumour cell lines. The study provides a good biochemical description of the BRD9-containing complex, which also contains some BAF complex components, and how this differs from other BAF-related complexes. The molecular biology describing what BRD9 actually does, or what the specific function of this complex might be, are much weaker with no definitive experiments performed to tell us what the complex does in healthy cells or why its absence causes SMARCB1-mutant tumour cells to die.

There is a general lack of explanation of what is being plotted in figures, size marker info on westerns, and information about replicate numbers. Quite often I have no idea what is being plotted in graphs. I also find it difficult to follow which cells are being used for which analyses. Were the RNA-seq and ChIP-seq experiments all performed in G401 cells with exogenous SMARCB1 expression? How many replicates were performed for each high-throughput sequencing experiment?

We thank Reviewer 2 for the constructive criticism regarding clarity. In retrospect we agree that this can be improved and we have now addressed this by revising figures and figure legends, which now include number of replicates in Figure 1D-E, G-H, as well as molecular weights. We have also clarified methods (ChIP-seq experiment: two biological replicates of BRD9 ChIP-seq, one replicate of SMARCA4, SMARCC1, H3K27Ac, and H3K4me3; RNA-seq experiment: two biological replicates for each sample; both RNA-seq and ChIP-seq experiment were performed in G401 cells (Figure 4 and Figure 5)). We also clarified cell lines used for immnunoprecipitation (Figure 2 B-C).

ChIP-seq: Class I sites are presumably those bound by this novel BRD9/SMARCA4 complex. What do Class II sites represent? The biochemical evidence so far indicates that BRD9 forms a novel complex with some BAF components as depicted in Figure 2D. These Class II sites, however, appear to be bound by BRD9 without SMARCA4 or SMARCC1. What do we make of these? Are the authors arguing that BRD9 also functions outside of this novel complex? The glycerol gradient gels do not support an idea that BRD9 can also function on its own.

We thank the reviewer for raising this interesting and important point. We both respond here and have revised the text of the manuscript to better communicate our findings on this data and analysis. Class II sites, which are preferentially located at H3K4me3 marked promoters, feature high BRD9 binding but little or no SMARCA4/SMARCC1 binding. Our glycerol gradient results from both G401

and 293T cells (**Figure 3E and F**) demonstrate that while much of BRD9 exists in large complexes with other SWI/SNF subunits, a portion also exists in much smaller fractions. This observation is consistent with a recent paper that detailed the modular assembly of SWI/SNF complex, which shows that a large amount of BRD9 exists in free form in 293T cells as well[1]. We have now revised the text to state: *the other half of BRD9 peaks (with little or no SMARCA4/SMARCC1 binding, Class II peaks) are preferentially enriched in H3K4me3-marked promoters (**Figure 4C-D**). This also suggests that BRD9 may function as free form or other smaller subcomplexes, which is consistent with glycerol gradient experiments from our data and others;*

The final section aims to identify "direct targets" of BRD9 using a published algorithm to try to work out what kinds of genes may be regulated by BRD9. They identify some genes and perform GO analyses, but in the end the conclusion is that BRD9 might somehow be involved in activating genes associated with apoptosis and in "additional biological functions". We really haven't learned much from this analysis. What are the authors trying to demonstrate here? If the aim is to learn why BRD9/SMARCB1 double null cells die, then it would be interesting to compare gene expression in both single mutants with a double mutant (e.g. a short time after loss of one of the proteins). If the question is rather "which genes show a transcriptional response to the loss of BRD9?" then it would be necessary to carry out an inducible deletion or depletion to distinguish primary transcriptional changes from secondary, tertiary, or adaptive transcriptional changes.

We thank the reviewer for this important point. In response we have clarified our questions/analyses. Additionally, we have expanded our analyses of the transcriptional changes from a single time point (4 days post BRD9 inactivation) to now include analyses at 1, 2, 3 and 5 days post-BRD9 inactivation to look at primary and secondary transcriptional changes.

We used our published algorithm BETA (Binding and Expression Target Analysis)[2] to: (1) examine whether BRD9 has activating or repressive, or both, function in regulating downstream gene expression; (2) identify BRD9 direct target genes as those with both BRD9 binding and demonstrating BRD9-dependent gene expression changes. Then we utilized GO (Gene Ontology) analyses to determine whether these direct target genes were enriched for previously identified functional categories. In using BETA, we found BRD9 binding was enriched at both activated and repressed genes rather than the static genes (Figure 5E). This demonstrates that BRD9 function is required for both activating and repressive functions in regulating gene expression. Given the distinct biochemical assemblies and binding of BRD9 in class I and class II sites we next analyzed these genes independently. As a reminder, Class I sites are those having both BRD9 and SMARCA4 binding while

Class II sites are those bound by BRD9 but showing little or no SMARCA4 or SMARCC1 binding. While there was substantial overlap in gene sets associated with Class I sites and Class II sites, there was stronger association of Class II sites with GO terms related to apoptosis and cell death while class I sites were preferentially associated with development (Figure 5F) thus raising the possibility of distinct functional roles for BRD9 when bound with and without canonical SWI/SNF subunits SMARCA4 and SMARCC1. We have now revised the manuscript to better communicate these points.

At Reviewer 2's suggestion, we performed RNA-seq at additional time points following CRISPR-Cas9 mediated deletion of BRD9. Our previous RNA-seq data was limited to a single time point, day 4 after BRD9 deletion, but we now present additional data for days 1, 2, 3, and 5 (**Supplementary Figure 7).** This new data reveals the transcriptional changes caused by BRD9 loss in a time-dependent manner. By comparing the distances of BRD9 binding to differentially expressed genes (both activated and repressed) and static genes (from Day 1 to Day 5), we found that differentially expressed genes are located closer to BRD9 binding sites than those that are static again suggesting that these represent direct effects of BRD9 (**Supplementary Figure 7C**). The transcription of select genes sets are differentially affected over time. For example, early on at Days 1 and 2 genes sets associated with chromatin structure are enriched, such as nucleosome assembly and gene silencing. Later on, at days 3 to 5, gene sets associated with downstream functions such as differentiation, development and apoptosis are enriched (**Supplementary Figure 7D-E**). Taken together, the updated results and synthesis (a combination of BRD9 ChIP-seq and RNA-seq following BRD9 deletion) demonstrate that BRD9 has both activating and repressive functions and regulates distinct pathways via direct targeting to enhancers and promoters. We added these new analysis to our revised manuscript.

Minor points

Minor comments:

1. Figure 1C: I think this is mislabelled: it looks like each panel has shRNA 1 and 2.

The reviewer is correct that Figure 1C was mislabeled. We have now corrected the labels.

2. Figure 1F: why are there residual protein bands in the G401 lanes if this is a knockout? Are these clonal lines?

G401-Cas9 cells were infected with lentiviral vector expressing sgRNA targeting BRD9, selected with puromycin, then plated as pooled populations for the proliferation assay. We have been unable to

generate BRD9 knockout clones as BRD9 loss ultimately proves lethal for RT cells (**Supplementary Figure 1**). Consequently, the residual bands represent cells within the pool that have escaped BRD9 targeting and we have been unable to eliminate them as they are positively selected for relative to BRD9-definient cells.

3.Lines 154-157 and Figure 2E: PBRM1 is widely distributed across the gradient gel, so it is incorrect to state that BRD9 and GLTSCR1 appear before PBRM1

The reviewer is correct and we have revised the text.

4. Line 165: The authors show that BRD9 is associating with other proteins individually in this assay, not that it forms a subcomplex (i.e. that it associates with all of them at the same time).

The reviewer is correct and we have revised the text. Indeed it is only the combination of IP and glycerol gradient results together that demonstrate BRD9 is found exclusively in a non-BAF, non-PBAF SWI/SNF sub-complex.

5. Line 173 and Figure 3D: There is almost no signal for GLTSCR1 on this blot. Is it expressed in these cells? It would be useful to have an input lane to demonstrate this. There is almost no signal in the input lanes of Supplementary Fig 3, again is this just not very well expressed in these cells?

Figure panel 3D is a glycerol gradient that compares the parental G401 RT cell line to the G401 line with BRD9 knocked out. GLTSCR1 is readily visible in the parental line but is lost from the BRD9-SWI/SNF subcomplex following BRD9 deletion. When BRD9 is present, GLTSCR1 occupies the same fractions as BRD9. Following BRD9 deletion, however, GLTSCR1 shifts to smaller fractions and exhibits a much weaker signal, producing the phenotype Reviewer 2 describes.

The input lane in **Supplementary Figure 3,** indeed shows that GLTSCR1 is somewhat weakly detected in the input but is heavily enriched in IP samples. The low input signal could be due to antibody quality, or possibly because GLTSCR1 is not highly expressed. These results are consistent (*Dykhuizen, 2018 JBC*)[4], who produced a similarly weak signal using the same GLTSCR1 antibody.

6. Figure 5A: Would be useful to include something that does show a change just for comparison, such as (presumably) unreconstituted G401 cells. Many of these lanes are overexposed so it would be difficult to detect modest changes

We did not detect any changes in histone modifications in G401 cells upon BRD9 loss. Consequently, other than the disappearance of BRD9 itself, we're unable to demonstrate a change in these cells. The findings from this Western blot are supported by the ChIP-Seq data in Panel 5B that shows no average change in K27ac signal upon BRD9 inactivation, while panel C provides further context by demonstrating that at individual genes BRD9 loss can result in either gain or loss of K27ac signal (averaging out to no change). We note that this result differs from our published results of inactivation of ARID1A, SMARCA4 and SMARCB1 (below).

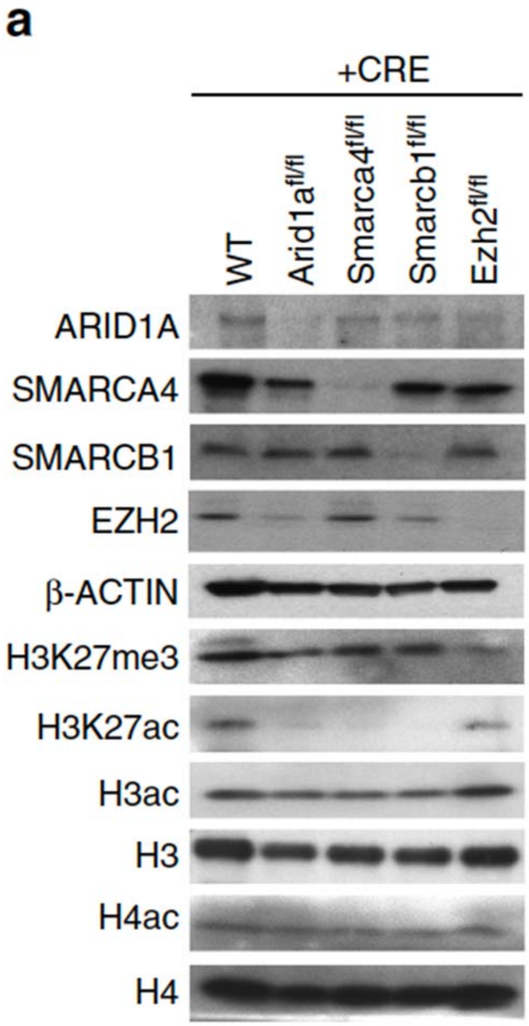

H3K27Ac is significantly decreased upon ARID1A, SMARCA4, SMARCB1 deletion in Mouse Embryonic Fibroblast (MEF) cells: from Alver *et al*[3]

7. Lines 226-227: I don't agree that the identification of DNA binding motifs for ETS family proteins at Class II sites, which are not bound by other SWI/SNF family members and occur overwhelmingly at promoters is "…consistent with a recent report that the SWI/SNF complex interacts with EWS-FLI1 and targets tumor-specific enhancers….".

The reviewer is correct and we agree that our original statement was too broad. We have now modified the language in the revised manuscript: "A recent report suggests that the SWI/SNF complex interacts with EWS-FLI1 and targets tumor-specific enhancers in Ewing sarcomas, raising the possibility a broader relationship between SWI/SNF complexes with ETS family transcription factors."

Reviewer #3 (Remarks to the Author):

Alpsoy and Dykhuizen (2018, JBC) previously demonstrated the existence of a sub-complex of BAF complexes which they termed "GBAF", for its unique incorporation of GLTSCR1/1L and BRD9, and the absence of core BAF components ARID1A/B, BAF57 and BAF47 (SMARCB1). In this manuscript, Wang et al., coming from an unbiased approach arising from the Achilles Project, report that SMARCB1-deficient rhabdoid tumors (RT) are differentially sensitive to the loss of BRD9 (Fig 1), suggesting that the GBAF complex might be involved in the RT malignancy.
The authors reproduce Dykhuizen's findings and confirm the existence of a BAF subcomplex defined by BRD9 and GLTSCR1. While this complex is present in both WT and SMARCB1-deficient RT cell lines, the authors propose but do not show conclusive proof that the GBAF complex dominates in SMARCB1-deficient RT cell lines. They further propose that this complex becomes essential in the absence or reduction of canonical SMARCB1-containing complexes, explaining the exquisite sensitivity of SMARCB1-deficient RT lines to BRD9-inhibition. Reduction of BRD9 causes the dissociation of complexes and subcomplexes, and re-expression of SMARCB1 induces re-assembly of canonical complexes.
In RT cell lines, the authors demonstrate that GBAF complexes have similar and distinct gene regulatory functions compared to canonical BAF complexes, and the transcriptional regulatory profile of BRD9 is consistent with a program that drives cell cycle and survival.
The findings presented in these paper are of interest due to the potential clinical utility of inhibiting BRD9 in RT based on its crucial role in BAF complexes. However, the authors have not demonstrated convincing evidence that BRD9 complex predominates in the absence of SMARCB1, which should be provided before the paper is suitable for publication.
Major comments:
1) The conclusion that SMARCB1 loss drives the formation of alternative BRD9 complexes hinges on

Figure 3E-F which are artificial manipulations of SMARCB1 expression. In my opinion, conclusive evidence that BRD9 complexes predominate in the absence of SMARCB1 include: a) glycerol gradients in WT and SMARCB1-mutant cell lines to see size redistribution of core subunits which in the SMARCB1-mutant lines presumably assemble with BRD9 complexes that are smaller. b) Immuno-depletion assays to show that in SMARCB1 mutant lines, BRD9 IP can immune-deplete core subunits which it assembles with to a greater extent than in WT lines.

2) Since the most interesting conclusion of the paper – that BRD9 inhibition might have therapeutic effect in RT and that this is not an in vitro phenomenon, the authors should attempt some xenograft experiments in the presence and absence of BRD9 knockdown. If the BRD9 degradation molecules are available, it would be even better to see if these have any efficacy as proposed in the concluding statement.

We're delighted that the reviewer feels that the findings are of interest due to potential clinical utility and we thank the reviewer for providing the thoughtful analysis. Per the Reviewer's suggestion, we have performed additional glycerol gradients in multiple cell lines in order to test the effects of addback of SMARCB1 to rhabdoid tumor cell lines and, conversely, the effects of SMARCB1 deletion from 293T cells.

First, we performed glycerol sedimentation assays in parental G401 rhabdoid tumor cells using our previously established SMARCB1 inducible re-expression system. BRD9, BAF and PBAF complex are modestly separated across gradient fractions in the absence of SMARCB1 (**Figure 2E – a new panel**). Notably, the SMARCA4 ATPase subunit overlaps primarily with fractions containing BRD9 rather than those occupied by BAF or PBAF subunits. Following SMARCB1 addback, as seen in the bottom half of the panel, there is a marked shift to the right of SMARCA4 relative to BRD9 and the amount of ARID1A is substantially increased, reflecting enhanced assembly of the BAF complex was noticeably enhanced.

To conversely evaluate the effects of SMARCB1 deletion we next conducted a second series of glycerol sedimentation assays in HEK293T cells and isogenic HEK293T SMARCB1-knockout cells. In parental cells, BRD9, BAF and PBAF complexes peak in distinct fractions (**Figure 2F – a new panel**). SMARCA4 broadly occupies fractions that overlap with BRD9, BAF and PBAF, though band intensity is greatest in those fractions associated with BAF. Deletion of SMARCB1 results in changes that are reminiscent of SMARCB1-deficient G401 RT cells. Specifically, BAF complex subunits ARID1A/B and DPF2 demonstrated reduced intensity and shift to smaller fractions indicating substantial effects of SMARCB1 loss in altering the integrity of the BAF complex. Further, SMARCA4 shifts from broad

overlap with BRD9, BAF, and PBAF-associated fractions to much more specific and tighter overlap with BRD9. The positioning of BRD9, however, appears unaffected by the loss of SMARCB1.

Finally, we performed a third series of glycerol sedimentation assays in HCT116 (SMARCB1-WT colon cancer) cells lines. These assays recapitulated the phenotype demonstrated in G401 SMARCB1 re-expressed and HEK293T SMARCB1-WT cells, in which BRD9, BAF and PBAF complexes are well separated and SMARCA4 overlaps across all three subtypes but predominately with BAF complex (**Supplementary Figure 2A**).

As suggested by the reviewer, we have also now performed BRD9 immuno-depletion in G401 cells without and with SMARCB1 re-expression (**Supplementary Figure 2B**). In parental G401 cells (without SMARCB1), BRD9 specifically depletes SMARCD1 and to a lesser degree SMARCA4, while as predicted it has no effect upon ARID2 (ARID2 is only in the PBAF sub-family of complexes and is not present in BRD9 complexes). Upon SMARCB1 re-expression the effect of BRD9 immunodepletion upon SMARCD1 and SMARCA4 is largely diminished. These results are consistent with those from the glycerol gradient. We don’t disagree with the reviewer that a BRD9 in vivo degradation could provide additional support. However, we do not have the dBRD9 molecule.

We sincerely thank the reviewer for the suggested experiment as the results from them have provided substantial additional support to our conclusions.

2. Minor comment:

1) Please refer to the reported subcomplex as GBAF since it appears to be the same sub-complex reported by Alpsoy.

We have added GBAF at the updated manuscript.

References:

1. Mashtalir, N. *et al.* Modular Organization and Assembly of SWI/SNF Family Chromatin Remodeling Complexes. *Cell* **175**, 1272–1288.e20 (2018).
2. Wang, S. *et al.* Target analysis by integration of transcriptome and ChIP-seq data with BETA. *Nat. Protoc.* **8**, 2502–2515 (2013).
3. Alver, B. H. *et al.* The SWI/SNF chromatin remodelling complex is required for maintenance of lineage specific enhancers. *Nat. Commun.* **8**, (2017).
4. Alpsoy, A. & Dykhuizen, E. C. Glioma tumor suppressor candidate region gene 1 (GLTSCR1) and its paralog GLTSCR1-like form SWI/SNF chromatin remodeling subcomplexes. *J. Biol. Chem.* (2018). doi:10.1074/jbc.RA117.001065

REVIEWERS' COMMENTS:

Reviewer #1 (Remarks to the Author):

I am satisfied with the authors' revised manuscript and support publication.

Reviewer #2 (Remarks to the Author):

This manuscript is much improved. Despite another paper coming out describing the synthetic lethality of BRD9 and SMARCB1 in RT, the current manuscript provides a good biochemical characterisation of BRD9 and its complexes, hence making important original contributions.

A few minor points remain:

Supplementary Figure 1A: please indicate replicate numbers and what the error bars represent.

Line 190: "the DUF3512 domain is both sufficient and necessary for BRD9 to form the subcomplex..." I don't still have the original manuscript, but this sounds like the statement the authors agreed to change. This assays shows the domain can form pairwise contacts, not that it forms a 'subcomplex.'

The time course expression data is a good addition and provides much more reliable information about BRD9-regulated genes. I just wonder whether there is any difference in behaviour of genes associated with Class I sites vs those associated with Class II sites? If so, it would be worth mentioning this in the text as it might give insights into the different functions of BRD9-containing complexes. If not, then perhaps best not mentioned!

Reviewer #3 (Remarks to the Author):

In the revised version of the manuscript, Roberts and colleagues have performed additional biochemical experiments to provide substantial evidence that BRD9 SWI/SNF subcomplexes predominate in the absence of SMARCC1. This has satisfied my concern regarding the true dominance of BRD9 complexes in the absence of SMARCC1.

In my opinion, the manuscript is now suitable for publication in Nature Communications.

We are glad that Reviewer #1 and #3 are satisfied with the revised manuscript and support publication; and Reviewer #2 raised only minor points.

Here we address those points:

Supplementary Figure 1A: please indicate replicate numbers and what the error bars represent.

Line 190: "the DUF3512 domain is both sufficient and necessary for BRD9 to form the subcomplex..." I don't still have the original manuscript, but this sounds like the statement the authors agreed to change. This assays shows the domain can form pairwise contacts, not that it forms a 'subcomplex.'

The time course expression data is a good addition and provides much more reliable information about BRD9-regulated genes. I just wonder whether there is any difference in behaviour of genes associated with Class I sites vs those associated with Class II sites? If so, it would be worth mentioning this in the text as it might give insights into the different functions of BRD9-containing complexes. If not, then perhaps best not mentioned!

1. We have now included the replicate numbers and clarified the error bars in Supplementary Figure 1A;
2. We have changed the language (Line 190) as suggested by the reviewer: "*we found that the DUF3512 domain is both sufficient and necessary for BRD9 interactions with other subcomplex subunits*".
3. While there was substantial overlap in gene sets associated with Class I sites and Class II sites suggesting a cooperative function, class II regions revealed more unique motifs, including 1) ETS family of transcription factors such as ETS1, ERG, and FLI1. This is also consistent with what we described in our main text Figure 5 and we have included this information.
